# Intrinsic ultrasmall nanoscale silicon turns n-/p-type with SiO_2_/Si_3_N_4_-coating

**DOI:** 10.3762/bjnano.9.210

**Published:** 2018-08-23

**Authors:** Dirk König, Daniel Hiller, Noël Wilck, Birger Berghoff, Merlin Müller, Sangeeta Thakur, Giovanni Di Santo, Luca Petaccia, Joachim Mayer, Sean Smith, Joachim Knoch

**Affiliations:** 1 Integrated Materials Design Centre, University of New South Wales, NSW 2052, Australia; 2 Laboratory of Nanotechnology, Dept. of Microsystems Engineering (IMTEK), University of Freiburg, 79110, Germany; 3 Research School of Engineering, The Australian National University, ACT 2601, Australia; 4 Institute of Semiconductor Electronics (IHT), RWTH Aachen University, 52074, Germany; 5 Ernst-Ruska Centre for Microscopy and Spectroscopy with Electrons, RWTH Aachen University, 52074, Germany; 6 Elettra Sincrotrone Trieste, Strada Statale 14 km 163.5, 34149 Trieste, Italy; 7 National Compute Infrastructure (NCI), The Australian National University, ACT 2601, Australia

**Keywords:** energy offset, impurity doping alternative, ultrasmall nanoscale silicon crystals, wires and devices

## Abstract

Impurity doping of ultrasmall nanoscale (usn) silicon (Si) currently used in ultralarge scale integration (ULSI) faces serious miniaturization challenges below the 14 nm technology node such as dopant out-diffusion and inactivation by clustering in Si-based field-effect transistors (FETs). Moreover, self-purification and massively increased ionization energy cause doping to fail for Si nano-crystals (NCs) showing quantum confinement. To introduce electron- (n-) or hole- (p-) type conductivity, usn-Si may not require doping, but an energy shift of electronic states with respect to the vacuum energy between different regions of usn-Si. We show in theory and experiment that usn-Si can experience a considerable energy offset of electronic states by embedding it in silicon dioxide (SiO_2_) or silicon nitride (Si_3_N_4_), whereby a few monolayers (MLs) of SiO_2_ or Si_3_N_4_ are enough to achieve these offsets. Our findings present an alternative to conventional impurity doping for ULSI, provide new opportunities for ultralow power electronics and open a whole new vista on the introduction of p- and n-type conductivity into usn-Si.

## Introduction

Impurity doping of silicon (Si) has been a key technique and prerequisite for Si-based electronics for decades [1]. Miniaturization in Si ultralarge scale integration (ULSI) became increasingly difficult as device features approached the characteristic lengths of dopant out-diffusion, clustering and inactivation [2]. The considerable broadening of dopant profiles from drain/source regions into gate areas persists [3]. Moreover, required ULSI transistor functionality and emerging applications of Si-nanocrystals (NCs) [4] unveiled additional doping issues: self-purification [5–6], suppressed dopant ionization [7–8] and dopant-associated defect states [8–9].

Modulation doping – i.e., doping of materials adjacent to semiconductors which then provide free carriers to the unperturbed semiconductor – was first used for group III–V semiconductor combinations such as GaAs/AlAs in the late 1970s [10]. Recently, Si modulation doping of adjacent dielectric layers based on nitrides [11] and oxides [12], in analogy to modulation doping of III–V semiconductors, were shown to be an alternative to conventional impurity doping.

It would be ideal to achieve electron- (n-) or hole- (p-) type conductivity in usn-Si without doping, thereby avoiding all dopant-related issues mentioned above. Such conductivity can be induced by an energy offset (Δ*E*) of the same electronic states (lowest unoccupied molecular orbital (LUMO) or highest occupied molecular orbital (HOMO)) between different regions of the same usn-Si system [13–14]. This concept eliminates doping altogether, leading to a lower inelastic carrier scattering rate and higher carrier mobility which allow for decreased heat loss and bias voltages in ULSI. Such properties enable Si-FET technology to work at even smaller structure sizes, potentially enabling Moore’s law to reach the Si-crystallization limit of ca. 1.5 nm [15].

In our present work, we prove by hybrid-density functional theory (h-DFT) simulations and synchrotron-based long-term ultraviolet photoelectron spectroscopy (UPS) that usn-Si indeed can have a massive Δ*E* of their electronic density of states (DOS) when embedded in SiO_2_ or Si_3_N_4_. We use further h-DFT results of a Si-nanowire (NWire) covered in SiO_2_ and Si_3_N_4_ to examine the device behaviour of an undoped Si-NWire FET based solely on CMOS-compatible materials (e.g., Si, SiO_2_, Si_3_N_4_) using the nonequilibrium Green’s function (NEGF) approach.

Following an explanation of the theoretical and experimental methods used, we turn to results for Si-NCs obtained from h-DFT. Here, we focus on the electronic structure of Si-NCs as a function of the embedding dielectric and its thickness of up to 3 monolayers (MLs). The latter dependence requires the use of NCs to keep the h-DFT computation effort practicable; NWires with more than 1 ML dielectric embedding are beyond the feasible computation effort at the level of accuracy we use. As an ultimate theoretical test, we present h-DFT results of two Si-NCs, one embedded in SiO_2_ and the other embedded in Si_3_N_4_, presenting the entire system under investigation within one approximant. An interface charge transfer (ICT) of electrons from the usn-Si volume to the anions of the embedding dielectric – nitrogen (N) or oxygen (O) – is at the core of the energy shift [14]. We explain the shift of usn-Si electronic states towards the vacuum level *E*_vac_ when embedded in Si_3_N_4_ and further below *E*_vac_ when embedded in SiO_2_ by the quantum chemistry of N and O with respect to Si. The next section contains experimental results, namely the thickness determination of embedded Si nanowells (NWells) by transmission electron microscopy (TEM) and the measurement of the highest occupied DOS over energy for Si-NWell samples embedded in SiO_2_ or Si_3_N_4_ by synchrotron-based long-term UPS. With this experimental confirmation of our h-DFT results, we present the concept of undoped Si-NWire field-effect transistors (FETs). We show further h-DFT results of a Si-NWire of 5.2 nm length and 1.4 nm diameter, terminated to 50% with 1 ML of Si_3_N_4_ (NH_2_ groups) and to 50% with 1 ML of SiO_2_ (OH groups). These h-DFT results deliver key input data to NEGF device simulations as a proof-of-concept for the undoped Si-NWire FET. A wealth of information on h-DFT accuracy as compared to experiment, details of UPS measurements and NEGF are contained in Supporting Information File 1.

## Experimental

### h-DFT material calculations

Hybrid-DFT calculations were carried out in real space with a molecular orbital basis set (MO-BS) and both Hartree–Fock (HF) and h-DFT methods as described below, employing the Gaussian03 and Gaussian09 program packages [16–17]. Initially, the MO-BS wavefunction ensemble was tested and optimized for stability with respect to describing the energy minimum of the approximant (variational principle; stable = opt) with the HF method using a Gaussian-type 3-21G MO-BS [18] (HF/3-21G). This MO wavefunction ensemble was then used for the structural optimisation of the approximant to arrive at its most stable configuration (maximum integral over all bond energies), again following the HF/3-21G route. Using these optimized geometries, their electronic structure was calculated again by testing and optimizing the MO-BS wavefunction ensemble with the B3LYP hybrid DF [19–20] and the Gaussian-type 6-31G(d) MO-BS which contains d-polarization functions (B3LYP/6-31G(d)) [21] to describe the strong polar nature of atomic bonds of Si to O and N. The root mean square (RMS) and peak force convergence limits for all atoms were 3 × 10^−4^ Ha/*a*_0_ (Hartrees per Bohr radius) or 80 meV/nm and 4.5 × 10^−4^ Ha/*a*_0_ or 120 meV/nm, respectively. Tight convergence criteria were applied to the self-consistent field routine. Ultrafine integration grids were used throughout. During all calculations, no symmetry constraints were applied to MOs. An extensive accuracy evaluation can be found in the Supporting Information File 1 of this article and elsewhere [13–14,22]. The approximants and MOs were visualized with GaussView 5 [23]. The electronic DOS were calculated from MO eigenenergies, applying a Gaussian broadening of 0.2 eV.

### Sample preparation

Samples comprising a Si_3_N_4_-embedded NWell were fabricated by plasma-enhanced chemical vapour deposition (PECVD) using SiH_4_+NH_3_+N_2_ for Si_3_N_4_ and SiH_4_+Ar for amorphous Si [24]. As substrates, n-type Si wafers (Sb doping, 5 to 15 × 10^−3^ Ω cm) of (111)-surface orientation underwent wet-chemical cleaning. After deposition the wafers were annealed in a quartz tube furnace for 1 min at 1100 °C in pure N_2_ ambient to induce Si crystallization. Subsequently, the samples were H_2_-passivated at 450 °C for 1 h. A 4.5 nm thick Si_3_N_4_ spacer layer served to suppress excited electrons from the Si wafer to interfere with electrons from the Si-NWell during UPS.

Samples comprising a SiO_2_-embedded NWell were processed by etching the top c-Si layer of an Si-on-insulator (SOI) wafer with 200 nm buried SiO_2_ (BOX) down to ca. 3 nm. The subsequent oxidation resulted in a 1.7 nm Si-NWell and 1.5 nm SiO_2_ capping.

Si reference samples were processed by etching a 5 to 15 × 10^−3^ Ω cm Sb-doped n-type (111)-Si wafer in buffered hydrofluoric acid, and the sample was immediately mounted under a N_2_-shower then swiftly loaded into the ultrahigh vacuum (UHV) annealing chamber.

All NWell samples were contacted via a lateral metal contact frame on the front surface which was processed by photolithographical structuring, wet-chemical mesa etching and thermal evaporation of Al. The reference Si-wafer was contacted directly on its front surface.

### Characterization

UPS measurements were carried out at the BaDElPh beamline [25] at the Elettra Synchrotron in Trieste, Italy, in top-up mode (310 mA electron ring current). All samples were subject to a UHV anneal for 90 min at 500 K to desorb water and air-related species from the sample surface prior to the measurements. Single scans of spectra were recorded over 12 h per NWell sample and subsequently added up for eliminating white noise. Scans for the Si-reference sample were recorded over 2 h and subsequently added up. All NWell samples were exited with a photon energy of 8.9 eV and a photon flux of 2 × 10^12^ s^−1^. The incident angle of the UV beam onto the sample was 50° with respect to the sample surface normal, and excited electrons were collected with an electron analyzer along the normal vector of the sample surface. The energy calibration of the UPS was realized using a tantalum (Ta) stripe in electrical contact to the sample as a work function reference. Further UPS-data of SiO_2_ and Si_3_N_4_ reference samples as well as UPS signal normalization are available in Supporting Information File 1.

All samples for TEM investigation were capped with a protective SiO_2_-layer to facilitate the preparation of cross sections by the focused ion beam technique using a FEI Strata FIB 205 workstation. Some samples were further thinned by means of a Fischione NanoMill. The TEM analysis of the cross sections was performed on a FEI Tecnai F20 TEM operated at 200 kV at the Central Facility for Electron Microscopy, RWTH Aachen University, and on the spherical aberration corrected FEI Titan 80-300 TEM operated at 300 kV at Ernst Ruska-Centre, Forschungszentrum Jülich [26].

In addition, the Si-NWell thickness was measured by ellipsometry. The thickness of the Si-NWells in Si_3_N_4_ (in SiO_2_) were measured using a Woollam M-2000 ellipsometer (ACCURION nanofilm ep4se ellipsometer). All thickness measurements confirmed the values obtained from TEM.

### NEGF device simulations

A homemade NEGF simulation program was used for simulating nanoscale device characteristics based on h-DFT results of Si-NWires. The simulations are based on a self-consistent solution of the Poisson and Schrödinger equations on a finite difference grid. A one-dimensional, modified Poisson equation is considered here that provides an adequate description of the electrostatics of wrap-gate nanowire transistors [27]. Buettiker probes, i.e., virtual contacts, are attached to each finite difference site in order to mimic inelastic scattering [28]. To this end, an additional self-consistent calculation of the quasi-Fermi level throughout the device is computed, ensuring that the net current flow into/out of each Buettiker probe is zero. The electrostatics within the gate underlap region has been taken into consideration with a conformal mapping technique that maps the underlap region to a parallel-plate capacitor and allows the extraction of a space-dependent effective oxide thickness that is used in this region. The “doping” due to the presence of the SiO_2_ coating is taken into consideration as a volume, active dopant concentration (see Supporting Information File 1); the presence of the Si_3_N_4_ layer underneath the gate is accounted for by an appropriate shift of the threshold voltage of the transistor (see Supporting Information File 1).

## Results and Discussion

### h-DFT calculations of embedded Si nanocrystals, fundamentals of energy offset

**Figure 1:**
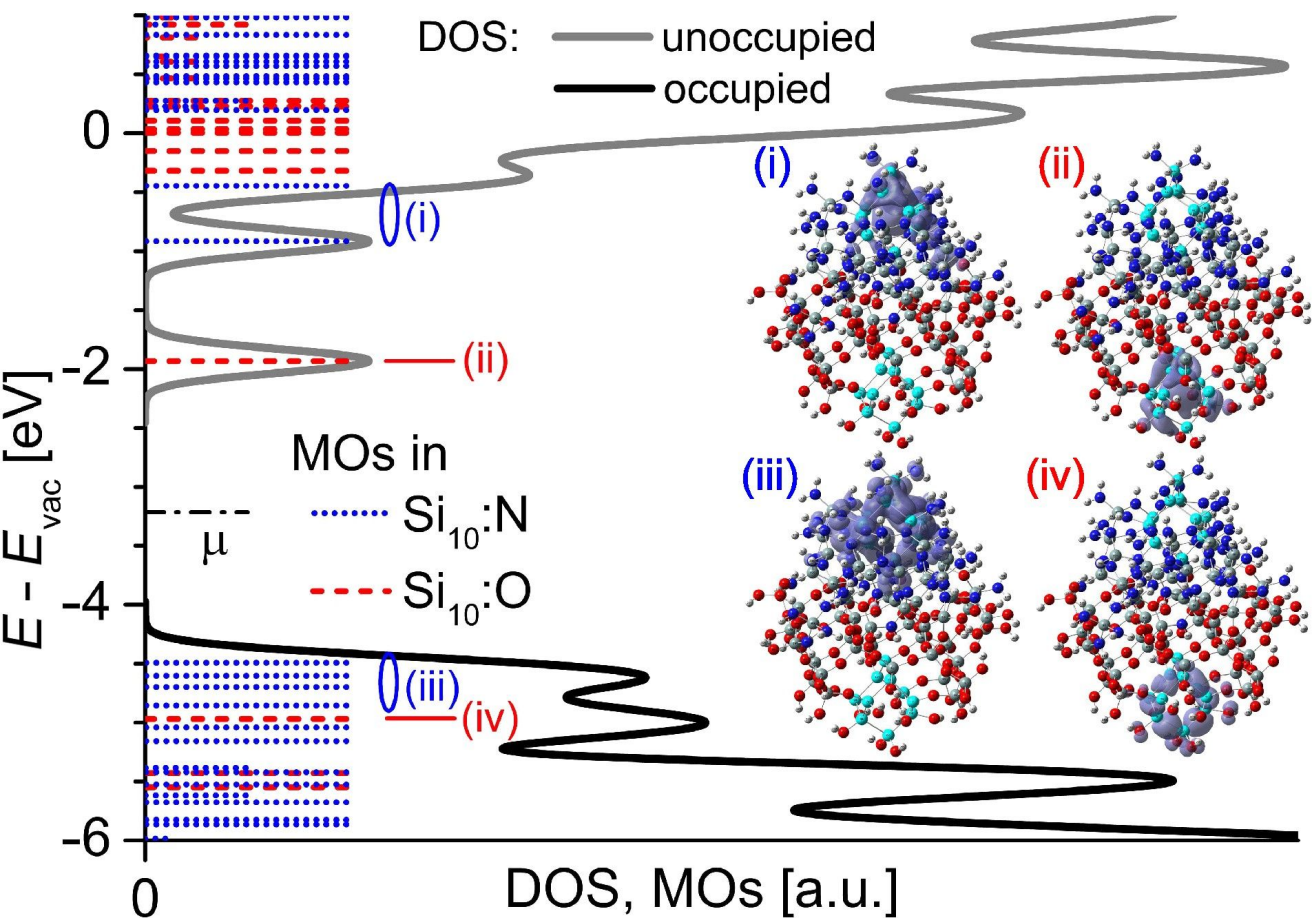
Energy offsets with SiO_2_- and Si_3_N_4_-embedding for one Si_10_-NC (0.8 nm size) embedded in SiO_2_ and the other Si_10_-NC embedded in Si_3_N_4_ within one approximant. The main graph shows the electronic DOS. MOs localized in Si_3_N_4_- (SiO_2_-) embedded Si-NC are shown in blue (red); the reduced length of the MOs corresponds to partial localization in Si_10_-NC, with the remainder of the MO being localized within the dielectric. The chemical potential of the entire approximant μ is shown as a dashed-dotted line. Graphs (i) to (iv) show iso-density plots (1 × 10^−3^ states/*a*_0_^3^ = 6.76 states/nm^3^) of frontier MOs marked by (i) to (iv) in the DOS plot. Si_10_-NCs are shown in cyan, Si in SiO_2_ and Si_3_N_4_ in grey, O in red, N in blue and H in white.

**Figure 2:**
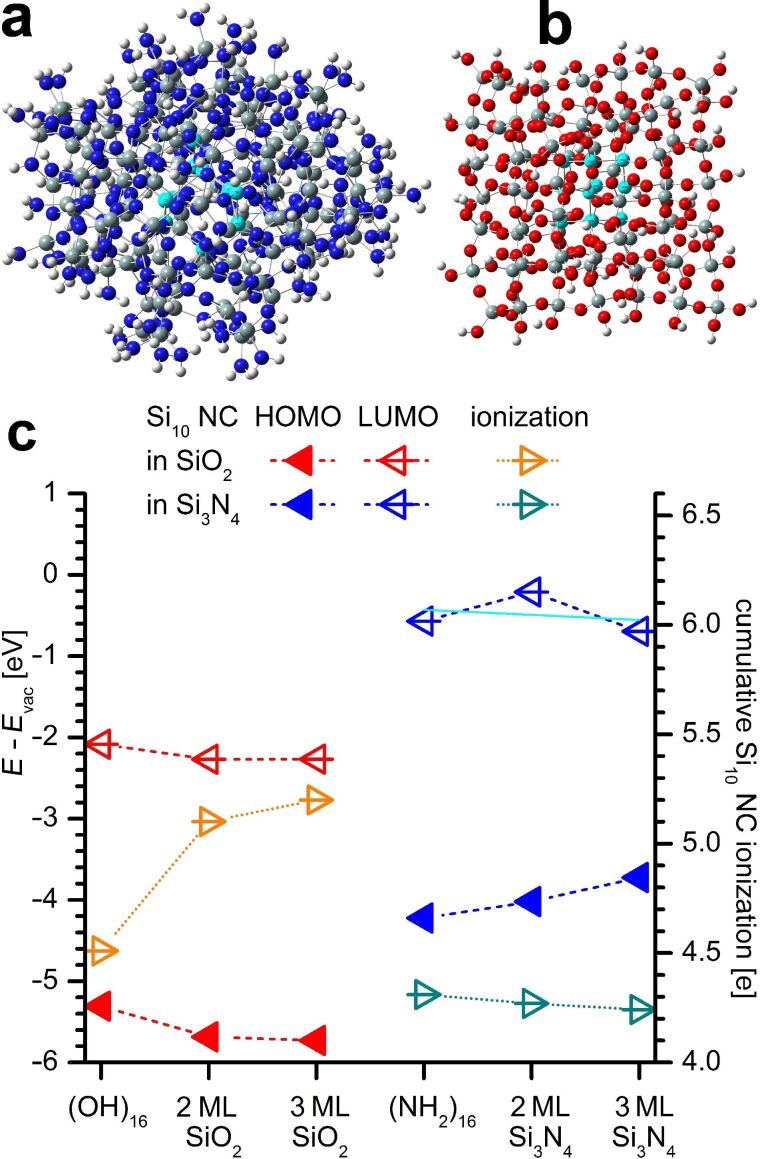
Evolution of energy offsets for SiO_2_- and Si_3_N_4_-embedded Si_10_-NCs (0.8 nm size) as a function of embedding SiO_2_- or Si_3_N_4_-thickness: (a) Si_10_-NC embedded in 3 ML Si_3_N_4_ after structural optimization. (b) Si_10_-NC embedded in 3 ML SiO_2_ after structural optimization. For atoms colors see Figure 1. (c) Evolution of HOMO and LUMO energies relative to vacuum energy *E*_vac_ (left scale) and total Si_10_-NC ionization (right scale) with increasing thickness of embedding dielectric. For SiO_2_-embedding, the ICT and the associated shift in HOMO and LUMO energies away from *E*_vac_ saturate quickly. For Si_3_N_4_-embedding, the HOMO energy shifts towards *E*_vac_. The LUMO energy shift varies around a constant value as shown by a linear fit to LUMO energies (cyan line) as a function of Si_3_N_4_ thickness. The positive NC ionization remains nearly unchanged. These features are due to the positive electron affinity *X* and the anionic nature of N, resulting in electron delocalization from the NC (ionization) without strong electron localization at N as is the case for O.

For evaluating the energy shift Δ*E* of the electronic DOS between usn-Si covered with SiO_2_ or Si_3_N_4_, we calculated two Si-NCs (Si_10_, 0.8 nm size) within one approximant; one NC is embedded in SiO_2_ and one NC resides in Si_3_N_4_ (Figure 1). We found earlier that – regarding DFT – Si_10_-NCs are the smallest NCs above the atomic limit below which Si-clusters behave as small molecules in the gas phase [13]. The frontier-OMOs exist within the Si_3_N_4_-embedded Si-NC (Figure 1, inset iii), while the frontier-UMO exists within the SiO_2_-embedded Si-NC (Figure 1, inset ii), with Δ*E* of the occupied frontier MOs of 0.5 eV and of 1 eV for the unoccupied frontier MOs between both NCs. These Δ*E* values are smaller when compared to individual embedded NCs (see Figure 2c and Supporting Information File 1) due to the inter-NC distance of merely 1 nm, accounting for some ICT convergence from Si NCs to SiO_2_ or Si_3_N_4_. From Figure 2c we see that an ICT saturation is evident for ≥2 ML SiO_2_. This saturation is less apparent when Si_3_N_4_ is applied as the embedding matrix. We explain this behaviour together with the Δ*E* by the quantum-chemical properties of Si, N and O.

Both anions, N and O, dominate electronic bonds to Si by delocalizing a substantial partition of Si valence electrons to form strong polar bonds [13], giving rise to ICT from usn-Si into the respective dielectric (SiO_2_, Si_3_N_4_) [14]. A high ionicity of bond (IOB) and strong negative electron affinity (*X*) of O result in a strong localization of Si-NC valence electrons. This localization corresponds to increased binding energies – the ICT shifts all MOs away from *E*_vac_. N is the only anionic element with a positive *X* [29] which is key for Δ*E* together with the smaller IOB of N to Si. Unlike O, the valence electrons delocalized from Si-NCs are *not* strongly localized at N due to its positive *X* and lower IOB to Si. Such delocalized MOs correspond to states with substantially lowered binding energy, yielding to a shift of MOs towards *E*_vac_. Accordingly, frontier-MOs of the Si_3_N_4_-embedded NC (Figure 1, insets i and iii) show stronger delocalization as compared to frontier-MOs of the SiO_2_-embedded Si-NC (Figure 1, inset ii and iv).

Table 1 summarizes the specific properties of Si, O and N relevant to the nature of ICT. The larger bond length of Si–N as compared to Si–O arguably contributes to electron delocalization, while the lower packing fraction of SiO_2_ is irrelevant in this respect due to strong electron localization at O. Both anions possess about the same ionization due to their IOB to Si together with N and O being trivalent and divalent, respectively. This finding is supported by the virtually identical NC ionization energy of fully NH_2_- vs OH-terminated Si-NCs (see Supporting Information File 1).

**Table 1:** Fundamental properties of N, O and Si: Ionization energy (*E*_ion_), electron affinity (*X*), electronegativity (EN), ensuing ionicity of bond (IOB) to Si and experimental values of characteristic bond lengths [29]. See also to Supporting Information File 1 for the latter.

element	*E*_ion_^a^ [eV]	*X* [eV]	EN^b^	IOB to Si [%]	*d*_bond_ to Si [nm]

N	14.53	+0.07	3.07	36	0.1743 (Si_3_N_4_)
O	13.36	−1.46	3.50	54	0.1626 (SiO_2_)
Si	8.15	−2.08	1.74	0	0.2387 (bulk Si)^c^

^a^Refers to first valence electron. ^b^Values after Allred and Rochow. ^c^With unit cell length of 0.5431 nm [30].

As will be shown experimentally in the next section, the resulting Δ*E* of the frontier-MOs induces an n-type (p-type) behaviour in usn-Si by SiO_2_-embedding (Si_3_N_4_-embedding). For the ICT, and thus the intensity of p- or n-type behaviour, the ratio of interface bonds to atoms forming the Si-NWell, -NWire or -NC is an important parameter [31]. It describes the amount of entities (Si atoms) to be ionized over a certain amount of transfer paths (interface bonds) and depends on the interface facet orientation of the usn-Si volume as well as on its surface-to-volume ratio.

### Sample characterization: TEM and synchrotron-based long-term UPS

**Figure 3:**
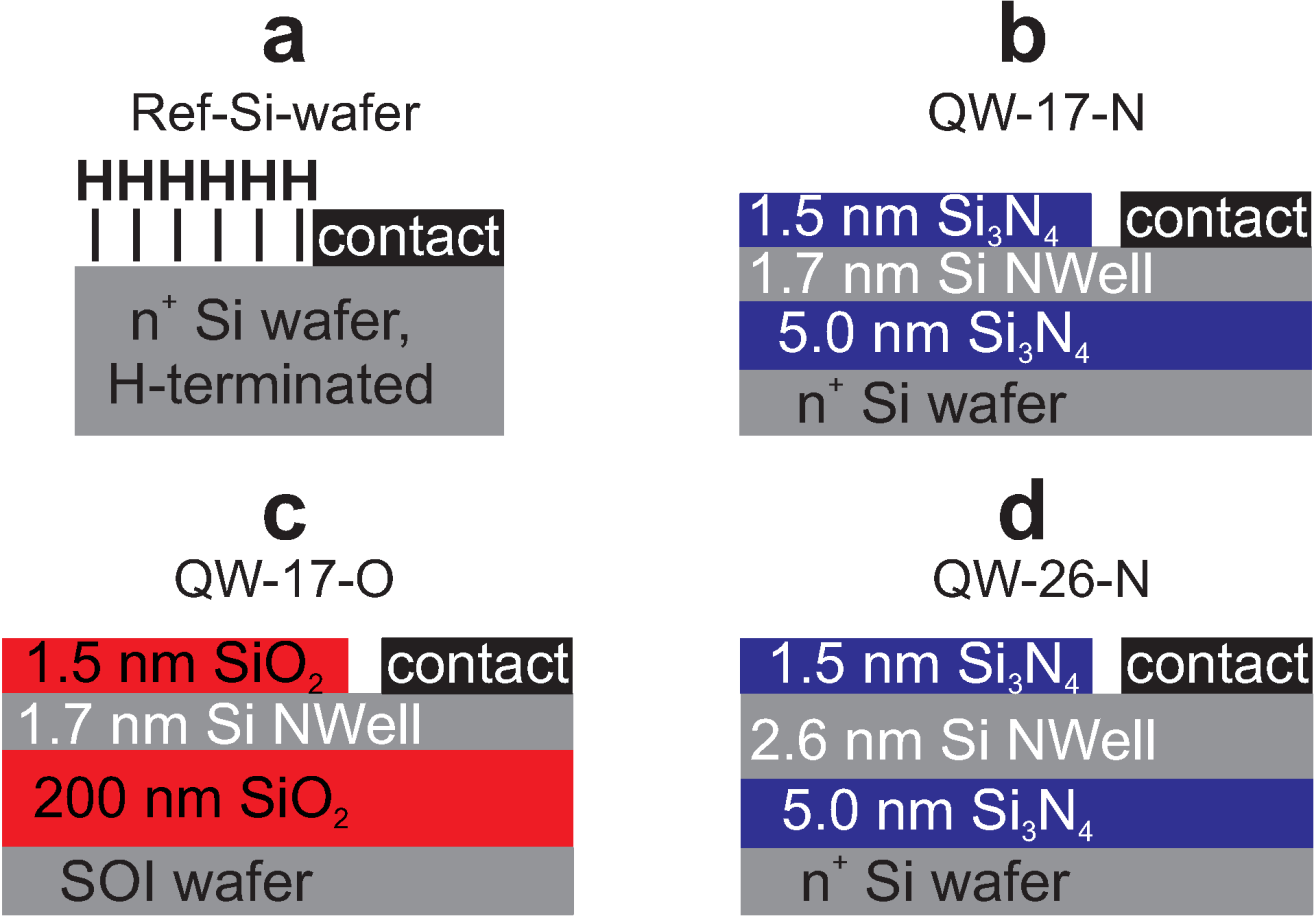
Structures of samples investigated by synchrotron UPS: (a) Si-reference, (b) 1.7 nm Si-NWell in Si_3_N_4_, (c) 1.7 nm Si-NWell in SiO_2_, (d) 2.6 nm Si-NWell in Si_3_N_4_. Sample codes are shown on top of each structure.

We experimentally verified our theoretical findings by characterizing samples comprising 1.7 nm and 2.6 nm thick Si-NWells embedded in SiO_2_ or Si_3_N_4_ together with a Si reference sample (Figure 3a–d) using synchrotron UPS.

**Figure 4:**
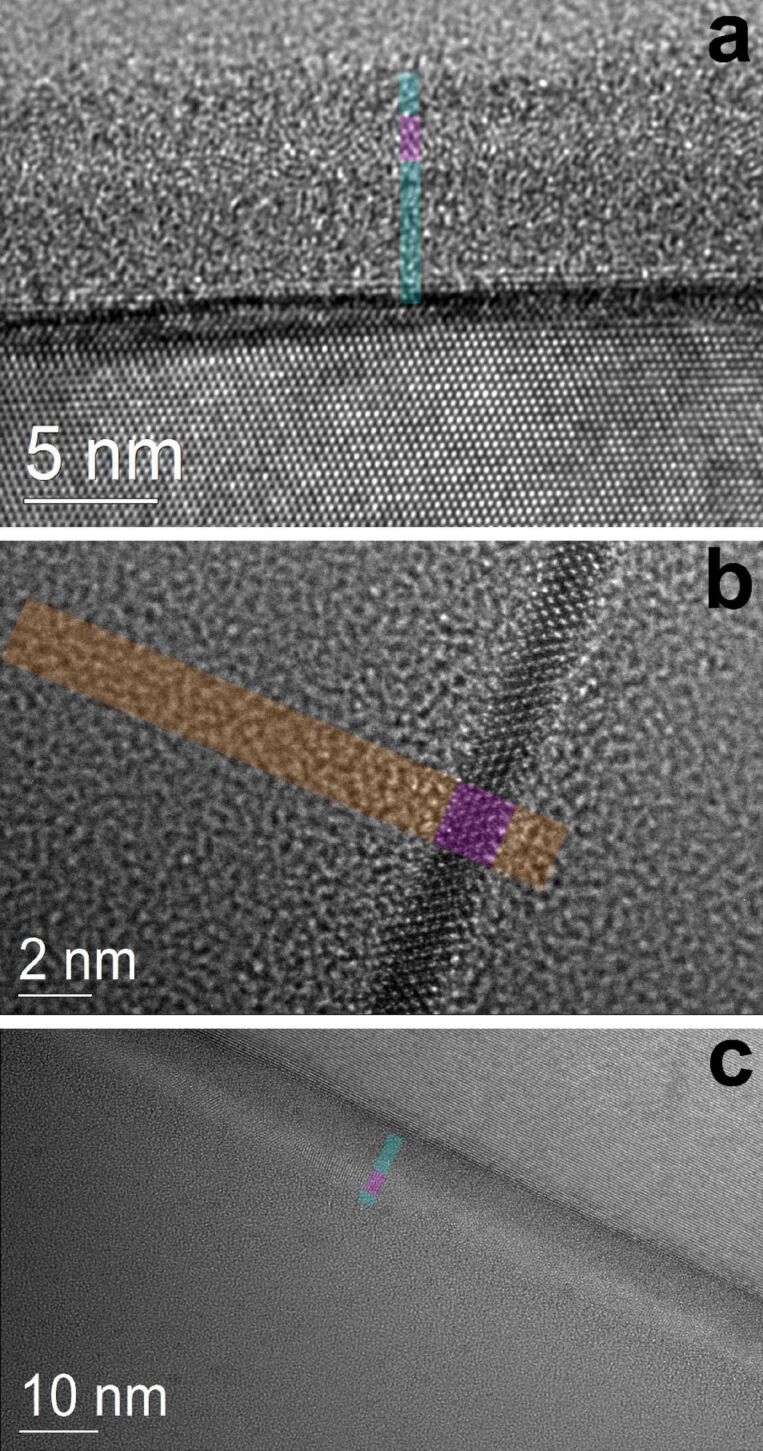
Cross-section HR-TEM images of samples QW-17-N (a), QW-17-O (b) and QW-26-N (c). Semi-transparent strips show layer thicknesses of Si_3_N_4_ (cyan), Si-NWells (magenta) and SiO_2_ (orange).

Figure 4a–c shows high-resolution cross-section TEM images of each NWell sample. Such ultrathin Si layers require long signal acquisition times in UPS due to the short mean free path of valence electrons excited above *E*_vac_[32] in compound with the small Si-volume probed. This is in particular true for Si-NWells embedded in Si_3_N_4_ as discussed in Supporting Information File 1.

**Figure 5:**
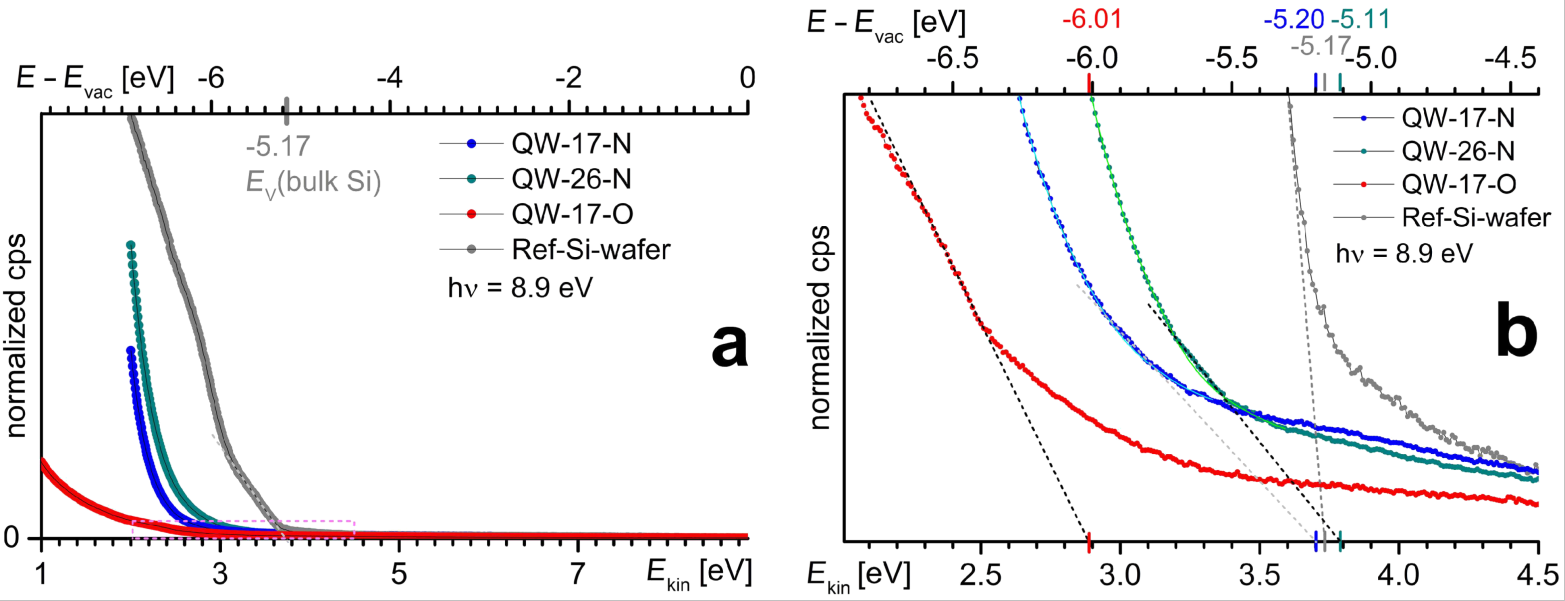
Experimental evidence of HOMO Δ*E* by synchrotron UPS: (a) scans of NWell samples and a hydrogen-terminated (111) Si wafer as a reference for the Si-NWells. The valence band edges of Si-NWells detected are located within the magenta lines and shown in (b). The bottom energy scales refer to electron kinetic energy up to UV photon energy. The top energy scale shows the energetic position of electrons relative to vacuum level with valence band edges and respective energy values as extracted from the spectra (dashed lines). The light green and cyan lines show the background fit of the amorphous Si_3_N_4_-matrix. The lower signal-to-noise ratio for Si-NWells embedded in Si_3_N_4_ as compared to SiO_2_ is comprehensively evaluated and discussed in Supporting Information File 1.

**Figure 6:**
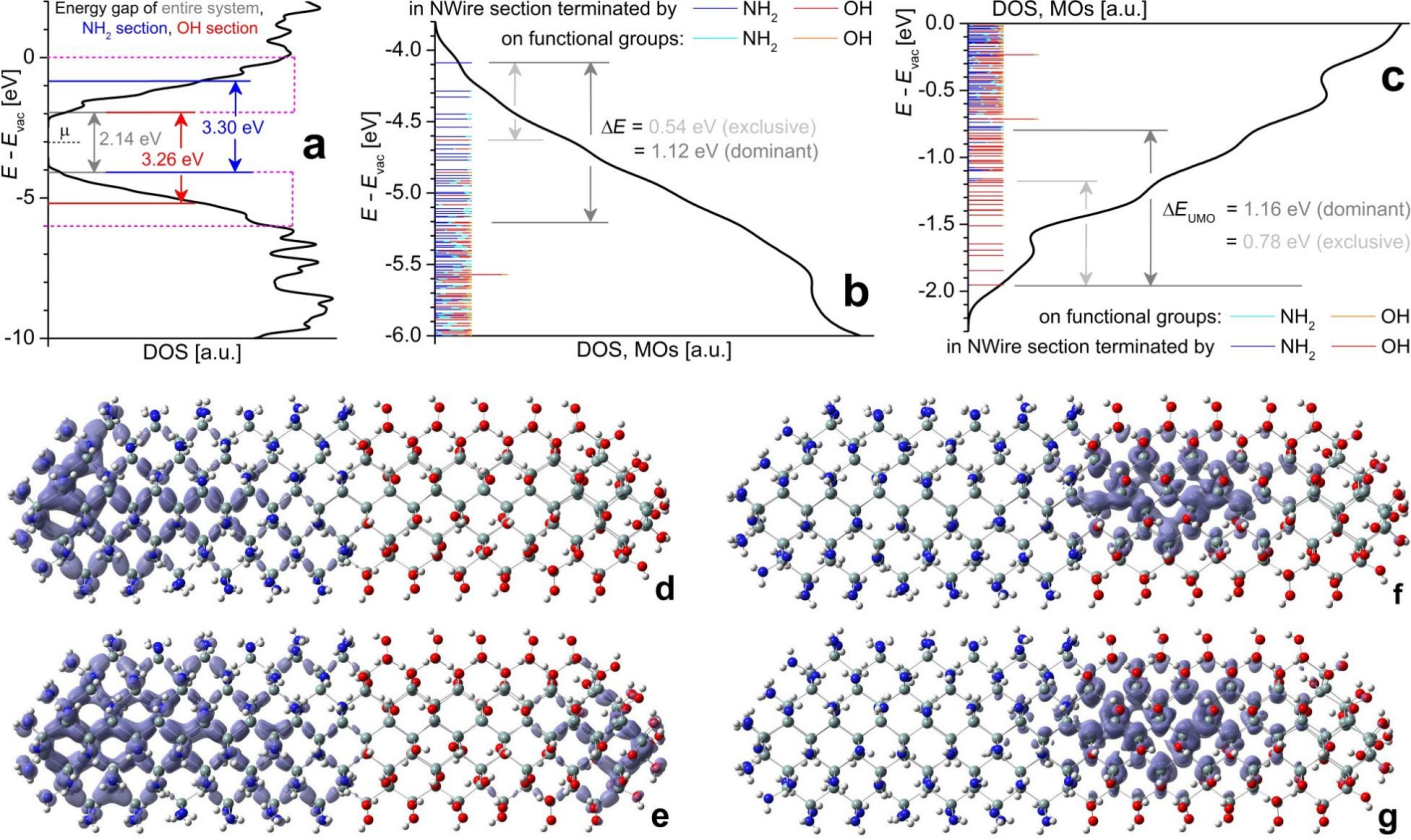
Electronic properties obtained by h-DFT for Si_233_(NH_2_)_87_(OH)_81_ NWire of 1.4 nm diameter and 5.2 nm length, terminated with NH_2_ on its left half emulating Si_3_N_4_-embedding and with OH on its right half emulating SiO_2_-embedding: (a) DOS over energy relative to vacuum level *E*_vac_. Red (blue) lines show HOMO–LUMO-gap of OH-terminated (NH_2_-terminated) NWire section. Global HOMO–LUMO gap shown in grey together with Fermi energy *E*_F_ for entire NWire. Magenta DOS sections are enlarged to show MO locations for (b) frontier-OMOs and (c) frontier-UMOs along with Δ*E* for exclusive and dominant MO location in the respective NWire section. (d–g) Si_233_(NH_2_)_87_(OH)_81_ approximant after structural optimization; for atom colours see Figure 1. The approximant is shown with the sum of frontier-MO densities ρ_MO_ = 
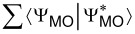
 as iso-density plots for: (d) frontier-OMOs exclusively located in the NH_2_-terminated NWire section (ρ_MO_ = 1 × 10^−3^ states/*a*_0_^3^ = 6.76 states/nm^3^), (e) frontier-OMOs dominantly located in the NH_2_-terminated NWire section (ρ_MO_ = 3 × 10^−3^ states/*a*_0_^3^ = 20.3 states/nm^3^). A slight distortion of atomic positions occurs at the OH-terminated end due to electrostatic forces, leading to a minor location of MOs otherwise exclusively residing in the NH_2_-terminated NWire section. This effect does not occur at NWire devices where SiO_2_ coverage is followed by a contact layer, see Figure 7. (f) Frontier-UMOs exclusively located in the OH-terminated NWire section (ρ_MO_ = 2 × 10^−3^ states/*a*_0_^3^ = 13.5 states/nm^3^), and (g) frontier-UMOs dominantly located in the OH-terminated NWire section (ρ_MO_ = 3 × 10^−3^ states/*a*_0_^3^ = 20.3 states/nm^3^). Values for ρ_MO_ are scaled to provide ρ_MO_ = 1 × 10^−4^ states/*a*_0_^3^ = 0.675 states/nm^3^ per MO.

UPS spectra are shown in Figure 5. The reference sample (Si-ref) yielded a valence band edge at the ionization energy *E*_ion_ = *E*_vac_ − 5.17 eV as known for bulk Si [33]. We obtained *E*_ion_ = *E*_vac_ − 6.01 eV for the 1.7 nm Si-NWell in SiO_2_ and *E*_ion_ = *E*_vac_ − 5.20 eV (*E*_vac_ − 5.11 eV) for the 1.7 (2.6) nm Si-NWell in Si_3_N_4_. The difference in ionization energy Δ*E*_ion_ between 1.7 nm Si-NWells in SiO_2_ and Si_3_N_4_ is 0.81 eV which clearly confirms our h-DFT calculations. For the 2.6 nm NWell embedded in Si_3_N_4_ we obtain a *E*_ion_ of 0.06 eV below the value of bulk Si (Figure 5b). The ICT may thus overcompensate quantum confinement and induce a negative Δ*E*_ion_ to bulk Si. The ICT impact length on Si-NWells can be related to Si-NWires and Si-NCs to scale 1/2/3 for NWells/NWires/NCs [14]. This relation explains why larger Δ*E* values for HOMOs and LUMOs are obtained for Si-NWires (Figure 6) as compared to Si-NWells (Figure 5b).

### Concept of undoped Si nanowire FETs

With the Δ*E* values of the usn-Si coated with SiO_2_ vs Si_3_N_4_ confirmed by synchrotron UPS, we now turn to its application to undoped ULSI Si devices.

NWire-FETs are a cornerstone of future ULSI technology development due to their excellent controllability by wrap-around gate architecture [34–35]. However, the ultrasmall NWire diameter required to guarantee the electrostatic integrity of the devices causes conventional doping to fail. Metal–Si contacts formed by, e.g., silicide formation [36] result in rather high Schottky-barriers at the source/drain-channel interfaces that deteriorate the switching behaviour and on-state performance.

### h-DFT calculations of Si nanowires relevant to devices

As we will show below, a Si-NWire with a combined SiO_2_-/Si_3_N_4_-coating can work as a highly scalable, high-performance and dopant-free metal-insulator-silicon (MIS) FET device. Using the same h-DFT methods as above, we computed the electronic properties of a Si_233_(NH_2_)_87_(OH)_81_ approximant manifesting a Si-NWire with 1.4 nm diameter and 5.2 nm length, whereby the two halves of this NWire are terminated with NH_2_ and OH groups, respectively. These functional groups correspond to 1 ML of the respective dielectric – NH_2_ groups to 1 ML Si_3_N_4_ and OH groups to 1 ML SiO_2_ (Figure 6).

Figure 6a shows the DOS around the HOMO–LUMO gap. We determined the location of the densities of all frontier-MOs, ρ_MO_ = 
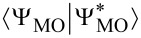
, within 2 eV from HOMO and LUMO. Frontier-OMOs are located within the NH_2_-terminated NWire section with a Δ*E* to corresponding MOs in the OH-terminated NWire section of ≈1.1 eV. Frontier-UMOs exist in the OH-terminated NWire section, whereby Δ*E* from the OH- to NH_2_-terminated NWire section is ≈1.2 eV. Again, the increased values of Δ*E* of respective frontier-MOs as compared to UPS results of Si-NWells confirm geometric effects [14].

### Undoped Si-NWire FETs

**Figure 7:**
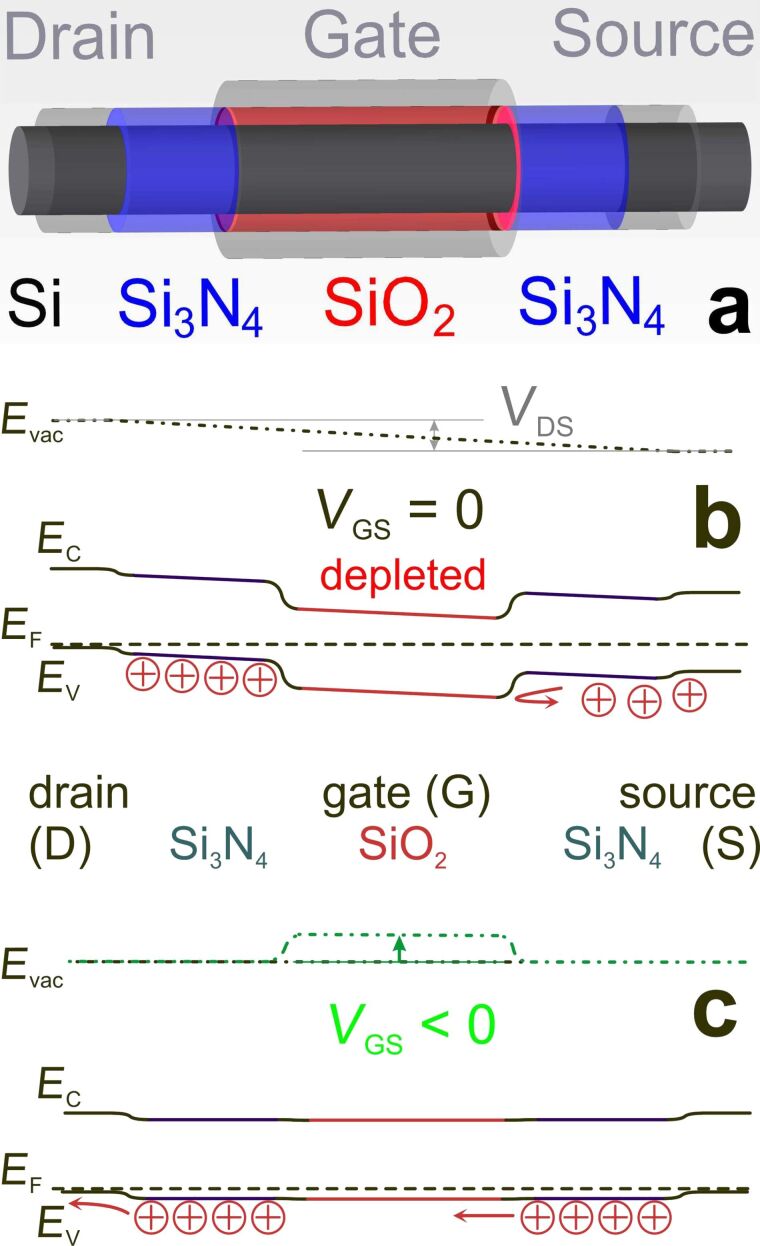
Concept of an undoped FET consisting of a Si-NWire with drain/gate (channel)/source regions covered by ultrathin Si_3_N_4_/SiO_2_/Si_3_N_4_: (a) physical layout shown for self-blocking p-channel FET. Schematic band diagram of such an FET shown for (b) zero and (c) negative gate bias relative to source voltage, resulting in a conductive channel by shifting the electronic Si-NWire states pinned by SiO_2_. Interchanging Si_3_N_4_ and SiO_2_ layers yields self-blocking n-channel FETs and thereby CMOS-compatibility. This concept is applicable to other Si nanostructures with a high surface-to-volume ratio like fin-FETs.

The electronic structure of the Si_233_(NH_2_)_87_(OH)_81_ NWire allows Δ*E* values to be established for NWire electronic devices with a combined SiO_2_-/Si_3_N_4_-coating such as an undoped self-blocking p-channel FET (Figure 7).

Using the Δ*E* value obtained from the Si_233_(NH_2_)_87_(OH)_81_ NWire approximant and above-described UPS results, we derive hole (*p*) and electron (*n*) densities. We obtain *p* = 5 × 10^19^ cm^−3^ (*n* ≈ 0 cm^−3^) for the Si_3_N_4_-coated NWire-regions (drain/source) and *p* = 71 cm^−3^ (*n* ≈ 0 cm^−3^) for the SiO_2_-coated NWire-regions (see Supporting Information File 1). These values will be used in the next section where results on NEGF device simulations are presented.

### NEGF device simulations

**Figure 8:**
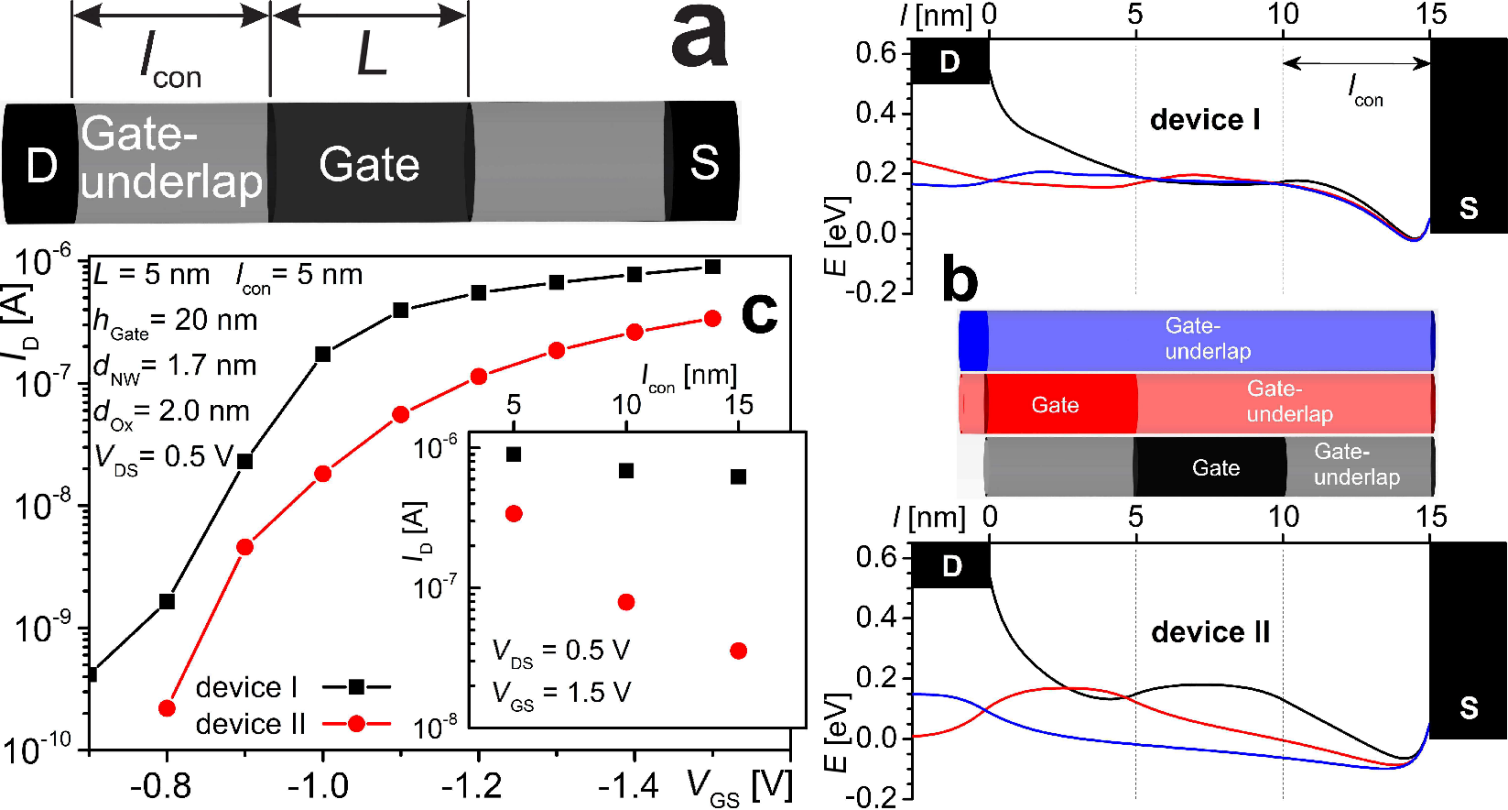
NEGF simulation results of undoped Si-NWire-FET illustrated in Figure 7: (a) gate-wrap-around Si-NWire FET showing parameters listed in graphs (b) and (c). (b) Valence band along the axis of device I (top, Si_3_N_4_-coated gate-underlap) and device II Si-NWire FET (bottom, entire Si-NWire SiO_2_-coated) in on-state-mode with *V*_GS_ = −1.2 V. The centre schematic shows the NWire-FET device gate-position and gate-underlap. Schottky-potential barriers build up although the same Schottky-barrier-height at the metal–Si interface at drain and source were chosen in both devices to examine the effectiveness of “doping” (Si_3_N_4_-coating) of underlap areas. A shift of the Schottky-barrier for device II due to workfunction mismatch of Ni to the valence band of the SiO_2_-coated Si-NWire would lead to a further massive deterioration of the on-state performance of device II. (c) Transfer characteristics of device type I (black) and II (red) for *V*_DS_ = 0.5 V, contact length *l*_con_ = 5 nm; the graph contains remaining parameters. The “doping” generated via ICT yields a substantially higher on-state performance in device I vs device II (no Si_3_N_4_-coated gate-underlap), an effect that becomes even more significant with increasing contact length *l*_con_, see inset. Hence, device II has low on-state performance and is prone to variability.

NEGF simulations were realized considering a 1.7 nm thick undoped Si-NWire MISFET with a channel length of *L* = 5 nm in a wrap-gate architecture placed between two metallic contacts (Figure 8a). The channel is insulated by a SiO_2_ layer, yielding an effective oxide thickness of 2 nm. The source/drain and the gate electrode are insulated from each other by an underlap region of length *l*_con_ where the NWire is covered with a 2 nm thick Si_3_N_4_ (device I) or SiO_2_ (device II) layer, resulting in dopant concentration equivalents as mentioned above. Ni source/drain contacts are considered to yield effective Schottky-barriers of −0.05 eV for hole-injection into the Si-NWire valence band.

Figure 8c shows drain-current versus gate-voltage characteristics of device I and II for an underlap of *l*_con_ = 5 nm. The SiO_2_ gate insulator yields a built-in potential that results in self-blocking FETs at *V*_GS_ = 0 V. Clearly, device I shows a substantially higher on-state performance, becoming even more obvious with increasing underlap region *l*_con_. The inset of Figure 8c displays the drive current at *V*_GS_ = −1.5 V, showing that device I exhibits very small current degradation with increasing *l*_con_ due to effective “doping” (Si_3_N_4_-coating) within the underlap region. In contrast, device II strongly depends on *l*_con_ with substantial drive current degradation if *l*_con_ increases. Device II only delivers an acceptable performance for *l*_con_ *<* 5 nm which ensues a very large parasitic capacitance and presents a challenge to ULSI processing. Moreover, any variation in *l*_con_ translates into a strong variability of drive current. This massive deterioration of device II is caused by the lack of “doping”, yielding a substantial increase in potential barriers (cf. Figure 8b) in particular at the gate-channel/gate-underlap interface and at the Ni–contact–Si interfaces, both depending on *l*_con_ (see Supporting Information File 1). Without the energy shift caused by Si_3_N_4_-coatings in source/drain, we obtain substantially higher Schottky-barriers for device II, resulting in severely deteriorated device performance. Our simulations underline the great importance of alternatives to conventional doping for increased performance of future ULSI transistors.

## Conclusion

We demonstrated quantitatively in theory and experiment that the intrinsic electronic properties of usn-Si can yield p- (n-) type behaviour by shifting the electronic DOS towards (away from) *E*_vac_ using ultrathin Si_3_N_4_- (SiO_2_-) coatings. The key parameters for this phenomenon are the electron affinities *X* of N and O together with their IOB and bond length to Si. Using NEGF device simulations we compared two undoped Si-NWire-FETs with SiO_2_- or Si_3_N_4_-coating in the source/drain regions and SiO_2_-coated gate area. We demonstrated that devices with Si_3_N_4_-coating exhibit substantially better on-state performance and strongly reduced dependence on the length of the source/drain regions, showing that high performance small-scale MISFETs can be realized using undoped ultrathin Si-NWires with a combined SiO_2_-/Si_3_N_4_-coating. Our findings open a whole new vista on Si-based ULSI operating at lower voltages and lower heat loss. Doping-related technological obstacles typical in CMOS technology are bypassed altogether, extending the potential of structural miniaturization down to the Si-crystallization limit of ca. 1.5 nm [15].

## Supporting Information

Supporting Information features the comparison of h-DFT results to experimental data, further information on the interface impact on Si nanocrystal electronic structure and its connection to quantum-chemical nature of N and O, details of UPS scans with further reference data, the derivation of charge carrier densities for nonequilibrium Green’s function (NEGF) transport simulation of undoped Si-nanowire MISFET devices and details on NEGF device simulations.

File 1 Further discussion and data of h-DFT, UPS, and NEGF simulations.
