# Supplementary material for: Intrinsic ultrasmall nanoscale silicon turns n-/p-type with SiO2/Si3N4-coating
Source: Beilstein J Nanotechnol. 2018 Aug 23;9:2255–64. doi: 10.3762/bjnano.9.210 (PMC6122087; doi:10.3762/bjnano.9.210)
Supplement: File 1 — Further discussion and data of h-DFT, UPS, and NEGF simulations. [file Beilstein_J_Nanotechnol-09-2255-s001.pdf]

Supporting Information

for

Intrinsic ultrasmall nanoscale silicon turns n-/p-type with SiO_2/Si_3N_4-coating

Dirk König*[1,2], Daniel Hiller[2,3], Noël Wilck[4], Birger Berghoff[4], Merlin Müller[5], Sangeeta Thakur[6], Giovanni Di Santo[6], Luca Petaccia[6], Joachim Mayer[5], Sean Smith[1,7] and Joachim Knoch[4]

Address: [1]Integrated Materials Design Centre, University of New South Wales, NSW 2052, Australia; [2]Laboratory of Nanotechnology, Dept. of Microsystems Engineering (IMTEK), University of Freiburg, 79110, Germany; [3]Research School of Engineering, The Australian National University, ACT 2601, Australia; [4]Institute of Semiconductor Electronics (IHT), RWTH Aachen University, 52074, Germany; [5]Ernst-Ruska Centre for Microscopy and Spectroscopy with Electrons, RWTH Aachen University, 52074, Germany; [6]Elettra Sincrotrone Trieste, Strada Statale 14 km 163.5, 34149 Trieste, Italy and [7]National Compute Infrastructure (NCI), The Australian National University, ACT 2601, Australia

Email: Dirk König - solidstatedirk@gmail.com

* Corresponding author

Further discussion and data of h-DFT, UPS, and NEGF simulations

Comparison of h-DFT Approximants to Experimental Data

We evaluate h-DFT calculations with identical compute algorithms as decribed in the main publication using approximants presenting pure dielectrics (SiO_2, Si_3N_4). Such approximants establish the base for a comparison with respective experimental bulk phase data. Si-NCs embedded in SiO_2 or Si_3N_4 do not present a good basis for comparison due to size distributions of Si-NCs and associated distributions in mechanical stress in experimental samples. Due to the wider distribution of bond lengths and angles, the calculation of amorphous SiO_2- and Si_3N_4-phases would require approximants in the range of at least 50,000 atoms to provide the statistical information (main value, standard deviation, skewness) for comparison with experiment. With the accurate h-DFT methods used here, the computation effort goes far beyond the feasible limit. We therefore resort to experimental data of the respective crystalline phase and show that our calculations closely match morphological and electronic experimental data of the respective dielectric bulk phase, underlining their accuracy and predictive power.

Morphology

The 3-21G Gaussian type MO-BS [1,2] used with the HF method delivers good accuracies for the structural optimization [3-6] and presents the optimum compromise between ultimate accuracy and tractable real-space computation for very large approximants such as the $Si_{165}(NH_2)_{100}$ (465 atoms) nanocrystal (NC) or the $Si_{233}(OH)_{81}(NH_2)_{87}$ NWire (718 atoms). For approximants with ground state HOMO–LUMO gaps above a certain threshold, the error in correlation forces is small for the occupied states due to their strong localization [7]. This threshold of the HOMO–LUMO gap for inorganic crystallites was found to be < 1.3 eV by results from numerous Si- and Ge-based approximants matching experimental band gap values [8]. On the other hand, exact exchange interaction is inherent to HF methods which is important for localized electron pairs as in atomic bonds [7].

For structural comparison we focus on $Si_{29}O_{76}H_{36}$ and $Si_{40}N_{86}H_{96}$ approximants presenting stoichiometric bulk phases of SiO_2 (α-quartz) and Si_3N_4 (α-phase), respectively, if we consider each H-atom as 1/4 Si. With this assumption, we get $Si_{38}O_{76} = 38$ SiO_2 formula units and

$Si_{64}N_{86} = 21\,\frac{1}{3}\,Si_3N_4$ formula units plus 2 N, resulting in $Si_3N_{4.03}$. Such approximants do not include structural deviations caused by local stress due to interfacing to Si-NCs or the other respective dielectric. Both approximants underwent structural optimization with the HF/3-21G and the B3LYP/6-31G(d) route. By computing approximants which contain one or two Si_{10} NCs with the HF/3-21G // B3LYP/6-31G(d) route, their results can be compared to each other as well as to earlier works of OH-terminated Si-NCs [4,5,9-11]. Both approximants are shown in Fig. S1.

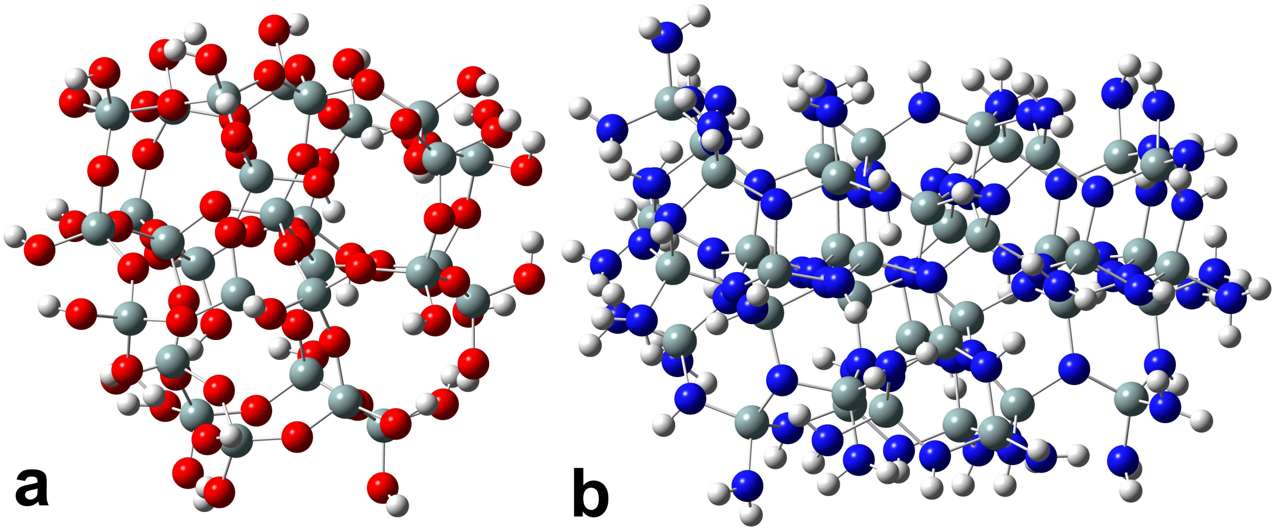

Figure S1: $Si_{29}O_{76}H_{36}$ and $Si_{40}N_{86}H_{96}$ approximants presenting SiO_2 (α-quartz) and α-Si_3N_4. The approximants are shown in the respective geometry after structural optimization with the B3LYP hybrid DF and a 6-31G(d) MO-BS. Structural differences to approximants optimized with the HF approach and a 3-21G MO-BS are difficult to spot solely by imaging, *cf.* Tables S1 and S2.

We compare the bond lengths $a_{\mathrm{Si-X}}$ (X = O, N) and bond angles $\alpha_{\mathrm{Si-X-Si}}$ of the two different optimization routes with experimental results and start with the $Si_{29}O_{76}H_{36}$ approximant describing SiO_2.

Table S1 shows the average and standard deviation of $a_{\mathrm{Si-O}}$ and $\alpha_{\mathrm{Si-O-Si}}$. Bonds to OH groups as outermost termination of the approximant were not included. These bond lengths have an average $a_{\mathrm{Si-OH}} = 1.620$ Å and a standard deviation of $\sigma(a_{\mathrm{Si-OH}}) = 0.013$ Å for the HF/3-21G optimization route. The higher level B3LYP/6-31G(d) optimization yielded $a_{\mathrm{Si-OH}} = 1.647$ Å and $\sigma(a_{\mathrm{Si-OH}}) = 0.013$ Å. The average bond lengths of Si to OH groups is only 0.5% and 0.7% larger as compared to $a_{\mathrm{Si-O}}$ of the HF/3-21G and B3LYP/6-31G(d) optimization, respectively. Referring

to bond geometry, this is a clear indication that the H termination of the outermost O atoms is an accurate approximation of an O–Si≡ bond.

Table S1: Average bond length $a_{\mathrm{Si-O}}$ and bond angles $\alpha_{\mathrm{Si-O-Si}}$ with their respective standard deviation $\sigma(a_{\mathrm{Si-O}})$ and $\sigma(\alpha_{\mathrm{Si-O-Si}})$ as a function of the optimization route for $Si_{29}O_{76}H_{36}$ (pure SiO_2 approximants). Bonds to OH groups were not included. Experimental and experimentally derived values are shown for comparison. XRD stands for X-ray diffraction.

**this work**				
$a_{\mathrm{Si-O}}$ [Å]	$\sigma(a_{\mathrm{Si-O}})$ [Å, %]	$\alpha_{\mathrm{Si-O-Si}}$ [°]	$\sigma(\alpha_{\mathrm{Si-O-Si}})$ [°, %]	optimization
1.612	0.0089, ±0.28	158.28	10.25, ±3.24	HF/3-21G
1.636	0.0106, ±0.32	143.49	10.93, ±3.81	B3LYP/6-31G(d)

**experimental and experimentally derived values**		
$a_{\mathrm{Si-O}}$ [Å]	$\alpha_{\mathrm{Si-O-Si}}$ [°]	remarks
1.626	143.6	vitreous silica; XRD combined with fluorescence excitation [12]
	155.0	exper. data from [12] corrected by structure refinement [13]
	155.6	idealized quartz model (undistorted SiO_4 tetrahedra) [14]

Table S1 shows that the SiO_2 approximant with B3LYP/6-31G(d) optimization has a geometry close to initial findings of experimental bond lengths and bond angles of alpha-quartz. The relative deviation from refined experimental values [13] is −0.62% and −7.43% for $a_{\mathrm{O-Si}}$ and $\alpha_{\mathrm{Si-O-Si}}$, respectively. For the lower level HF/3-21G optimization, the relative deviation of $a_{\mathrm{O-Si}}$ from experiment is −0.86%. The main value of the respective $\alpha_{\mathrm{Si-O-Si}}$ is just 2.1% off the corrected experimental value. Moreover, these bond angles shows a proximity to idealized quartz derived by Hobbs, Jesurum and Berger [14], with a relative deviation of 1.72%. The counter-intuitive finding of the rather simple HF/3-21G optimization route yielding to higher structural accuracy as compared to the more elaborate B3LYP/6-31G(d) computation route is nested in the exact description of exchange interaction with the HF method, a dominant feature of atomic bonds formed by electron pairs strongly localized in binding orbitals.

We now look at the $Si_{40}N_{86}H_{96}$ approximant presenting Si_3N_4. Alike to the $Si_{29}O_{76}H_{36}$ approximant above, we split bonds and angles involving N into similar fractions as a function of H attachment. This classification yields to three fractions of bond lengths – $a_{\mathrm{N-Si}}(>\mathrm{N}-)$, $a_{\mathrm{N-Si}}(-\mathrm{NH}-)$, $a_{\mathrm{N-Si}}(-\mathrm{NH_2})$ – and two fractions of bond angles with N as the central atom – $\alpha_{\mathrm{Si-N-Si}}(>\mathrm{N}-)$,

$\alpha_{Si-N-Si}(-NH-)$. Main values and standard deviations of all $a_{N-Si}(>N-)$ and $a_{N-Si}(-NH-)$ as well as all $\alpha_{Si-N-Si}(>N-)$ and $\alpha_{Si-N-Si}(-NH-)$ relevant for pure Si_3N_4 are listed in Table S2.

Table S2: Average bond length a_{Si-N} and bond angles $\alpha_{Si-N-Si}$ with their respective standard deviation $\sigma(a_{Si-N})$ and $\sigma(\alpha_{Si-N-Si})$ as a function of the optimization route for $Si_{40}N_{86}H_{98}$ (pure Si_3N_4 approximants). Data from calculations refer to all $^{Si}_{Si}{>}N{-}Si$ and $Si{-}NH{-}Si$ bonds and resulting bond angles over $^{Si}_{Si}{>}N{-}Si$ and $Si{-}NH{-}Si$. Experimental values are shown for comparison.

this work				
a_{Si-N} [Å]	$\sigma(a_{Si-N})$ [Å, %]	$\alpha_{Si-N-Si}$ [°]	$\sigma(\alpha_{Si-N-Si})$ [°, %]	optimization
1.757	0.0163, ±0.46	120.24	4.28, ±1.78	HF/3-21G
1.755	0.0152, ±0.43	119.69	4.86, ±2.03	B3LYP/6-31G(d)

experimental values		
a_{Si-N} [Å]	$\alpha_{Si-N-Si}$ [°]	remarks
1.75		amorphous Si_3N_4 deposited by CVD [18]
1.73	121	amorphous Si_3N_4 deposited by CVD [19]
1.75		amorphous Si_3N_4 deposited by CVD [20]

It is well known from Si_3N_4 deposition that a considerable amount of H may be incorporated into Si_3N_4 usually introduced by ammonia (NH_3) as a pre-cursor, providing more reactive N radicals after plasma decomposition. This ability to incorporate H and its sub-sequent release at elevated temperatures is often exploited for chemical passivation of Si interfaces in silicon solar cell processing [15,16]. Hence, bond terminations of N by H in the $Si_{40}N_{86}H_{96}$ approximant are more than an necessity to terminate outermost bonds and keep the Si_3N_4 stoichiometric if we count every H atoms as 1/4 of Si. Interestingly, it was found that a H incorporation as high as 20 mol % into SiN_x only results in a 0.2 eV decrease of the bandgap if the ratio of N/Si is kept roughly stoichiometric (with the deviation being compensated by H incorporation), i.e., $x = 1.3 \ldots 1.4$ [17].

The bond lengths of NH_2 groups to Si have an average value of $a_{Si-NH_2} = 1.742$ Å and a standard deviation of $\sigma(a_{Si-NH_2}) = 0.0073$ Å for the HF/3-21G optimization route. The higher level B3LYP/6-31G(d) optimization yielded $a_{Si-NH_2} = 1.743$ Å and $\sigma(a_{Si-NH_2}) = 0.0122$ Å. The average bond lengths of Si to NH_2 groups is 1.2% and 1.0% smaller as compared to $a_{Si-N}(>N-)$ of the HF/3-21G and B3LYP/6-31G(d) optimization, respectively. We can consider the H termination of outermost N atoms as a reasonably accurate approximation of an N–Si≡ bond, whereby the H-termination of N atoms has no significant effect on the Si_3N_4 band gap. This minor influence is due to the bonding-

antibonding MO splitting being significantly bigger for the H–N bond than the Si–N bond. The situation improves greatly for the bond lengths of –NH– groups. We get $a_{\mathrm{Si-NH-Si}} = 1.749$ Å and a standard deviation of $\sigma(a_{\mathrm{Si-NH-Si}}) = 0.0133$ Å for the HF/3-21G optimization route. The higher level B3LYP/6-31G(d) optimization yielded $a_{\mathrm{Si-NH-Si}} = 1.745$ Å and $\sigma(a_{\mathrm{Si-NH-Si}}) = 0.0135$ Å. The deviation of $a_{\mathrm{Si-NH-Si}}$ from the average of the experimental values in Table S2 – $a_{\mathrm{N-Si}} = 1.743$ Å – is merely 0.31% for the HF/3-21G route (0.11% for B3LYP/6-31G(d) route). This is an indication that NH_2 groups do not exist in real Si_3N_4 and virtually all incorporated H bond to N is attached to –N– bridging two Si atoms. We can derive from this finding that for $Si_3N_{4.2}$:H [17], about 20% of N atoms exists in this –NH– bridging configuration. The values of Si−N bond lengths for fully Si-terminated N are 1.07% (1.01%) larger for the HF/3-21G (B3LYP/6-31G(d)) route as compared to the average of experimental values of 1.743 Å obtained from data in Table S2. For the cumulative value of bond lengths $a_{\mathrm{Si-N}_{all}}$, we do not consider the bonds $\mathrm{Si-NH_2}$ as they do not appear to exist in Si_3N_4 and Si_3N_x:H. We then get $a_{\mathrm{Si-N}_{all}} = a_{\mathrm{Si-NH-Si}} + a_{\mathrm{Si-N}}(>\mathrm{N}-) = 1.757$ Å for the HF/3-21G route (1.755 Å for the B3LYP/6-31G(d) route), see Table S2. Such values are 0.78% (0.67%) bigger as compared to the average of the experimental values in Table S2 = 1.743 Å, showing a high accuracy in structural optimization. Here, the higher level B3LYP/6-31G(d) optimization route is slightly more accurate regarding bond lengths.

Results on Si–N–Si bond angles favour the HF-3-21G route when compared to the experimental value of $\alpha_{\mathrm{Si-N-Si}} = 121.0°$; the deviation is $-1.78°$ or -0.98% ($-2.21°$ or -1.83% for the B3LYP/6-31G(d) route). If angles of the form Si−NH−Si are included into the analysis, we get $\alpha_{\mathrm{Si-N-Si}} = 120.24 \pm 2.14°$ for the HF/3-21G route ($\alpha_{\mathrm{Si-N-Si}} = 119.69 \pm 2.43°$ for the B3LYP/6-31G(d) route), whereby the $\pm$ tolerance stands for the standard deviation, *cf.* Table S2. This cumulative value diminishes the error to experiment down to $-0.76°$ or -0.63% ($-1.31°$ or -1.08% for the B3LYP/6-31G(d) route). In both cases, the calculated values of $\alpha_{\mathrm{Si-N-Si}}$ are very close to experiment, with the HF/3-21G route having an edge over the more costly B3LYP/6-31G(d) route. Considering angles of the Si–NH–Si bonds in addition to $^{\mathrm{Si}}_{\mathrm{Si}}$>N–Si bonds is the more realistic option due to the existence of significant amounts of H in CVD-deposited Si_3N_4, see above.

Electronic Structure

Approximants were calculated using the HF/3-21G // B3LYP/6-31G(d), the B3LYP/6-31G(d) // B3LYP/6-31G(d) and the B3LYP/6-31G(d) // B3LYP/6-31+G(d) route. The latter was chosen in order to elucidate the impact of the different optimization methods onto the electronic structure. It also enables us to evaluate the impact of the diffuse primitive Gaussian functions on the electronic structure.

In real-space DFT calculations, the exact calibration to an absolute energy scale (the vacuum level E_{vac}) is known to be ambiguous [21]. Nevertheless, *relative changes of energy values such as band offsets or fundamental energy gaps between approximants with different interface termination are accurate within the same computation route*. Therefore, the results of the production runs obtained with the HF/3-21G(d)//B3LYP/6-31G(d) route are accurate apart from a constant offset for all MO energies and hence trustworthy. As a consequence, all energy-dependent results are accurate within the same real-space DFT//MO-Basis-Set method and can be compared with experimental data on a relative base such as ΔE between SiO_2- and Si_3N_4-coated Si-NWire-sections or electronic gaps [22]. For the $Si_{29}O_{76}H_{36}$ approximant, we see that the band gap is underestimated for all DFT methods used. However, experimental values of E_{gap} of thin thermal SiO_2 layers formed by oxidizing Si wafers vary considerably from 8.0 to 8.9 eV, while bulk forms of SiO_2 have even lower values, see to bottom of Table S3. The rather wide range of experimental values for the fundamental gap of SiO_2 add to the complexity of a quantitative evaluation of DFT results against experiment. The best result is obtained once again with the HF/3-21G // B3LYP/6-31G(d) route since it gets closest to the range of experimental E_{gap} values.

For Si_3N_4 we presume an experimental value of $E_{gap}(Si_3N_4) = 5.4$ eV which results from the average of experimental findings in Table S4. The DFT results of the $Si_{40}N_{86}H_{98}$ approximant show that the HF/3-21G // B3LYP/6-31G(d) route is slightly more accurate (rel. error $= +10.1\%$) as compared to the B3LYP/6-31G(d) // B3LYP/6-31G(d) route (rel. error $= +11.4\%$). While the better accuracy in the electronic structure for a lower level optimization may be counter-intuitive, we note that the advantages of the HF method for structural optimization as discussed above have far-reaching con-

Table S3: HOMO and LUMO levels E_{HOMO}, E_{LUMO} and fundamental energy gap E_{gap} as function of the optimization route for the $Si_{29}O_{76}H_{36}$ (pure SiO_2) approximant, followed by respective experimental E_{gap} values, sub-divided into bulk samples (top) and thin layers grown on Si (bottom). Abbreviations for characterization techniques: X-ray photoelectron spectroscopy (XPS); soft X-ray emission spectroscopy (SXES); ultraviolet-absorption (UV-A), -reflection (UV-R); electron energy loss spectroscopy (EELS), photo-conductivity (PC), photo-injection (PI).

**SiO_2, this work**			
E_{HOMO} [eV]	E_{LUMO} [eV]	E_{gap} [eV]	optimization// electronic structure (stable=opt)
−7.634	−0.033	7.601	HF/3-21G // B3LYP/6-31G(d)
−8.014	−0.462	7.552	B3LYP/6-31G(d) // B3LYP/6-31G(d)
−8.393	−1.320	7.073	B3LYP/6-31G(d) // B3LYP/6-31+G(d)

**experimental values**	
E_{gap} [eV]	remarks
7.5	optical absorption; synthetic quartz crystal [23]
8.5	optical absorption; fused quartz [23]
7.8 … 5.6	XPS, SXES, UV-A & -R, EELS, PC, PI; α-quartz [24]
8.3 … 7.6	see previous line; OH-free SiO_2 (Cab-O-Sil, Corning Inc.) [24]
8.8	exciton generation, induced fixed charge; 200 nm dry thermal SiO_2 on Si [25]
8.9 ± 0.2	PC; 500 nm thermal SiO_2 on Si [26]
8.9	UPS, AES, EELS; UHV, Si surface exposed to minute amounts of O_2 [27]
8.0 ± 0.2	PC; free standing 58 nm thermal SiO_2 on {001} Si by Si etch-back [28]

sequences into electronic structure calculations. The use of primitive diffuse Gaussian functions ("+") on all but H atoms in the 6-31+G(d) MO-BS decreases E_{gap} again, providing a slightly better accuracy (rel. error = −8.83%) than the HF/3-21G // B3LYP/6-31G(d) route. Unfortunately, the 6-31+G(d) MO-BS is not a feasible choice for systems above ca. 150 atoms due to numerical divergence and stability and hence no option for the approximants calculated in the main publication.

Table S4: HOMO and LUMO levels E_{HOMO}, E_{LUMO} and fundamental energy gap E_{gap} as function of the optimization route for the $Si_{40}N_{86}H_{98}$ (pure Si_3N_4) approximant, followed by respective experimental E_{gap} values. RF stands for radio frequency.

Si_3N_4, **this work**			
E_{HOMO} [eV]	E_{LUMO} [eV]	E_{gap} [eV]	optimization// electronic structure (stable=opt)
−4.701	1.245	5.946	HF/3-21G // B3LYP/6-31G(d)
−5.160	0.989	6.149	B3LYP/6-31G(d) // B3LYP/6-31G(d)
−5.637	−0.714	4.923	B3LYP/6-31G(d) // B3LYP/6-31+G(d)

**experimental values**	
E_{gap} [eV]	remarks
5.5	amorphous Si_3N_4 deposited by RF glow discharge [29]
5.3	amorphous $Si_3N_{4.2}$:H[a] deposited onto amorphous Si by RF glow discharge [17]

[a]Corresponds to 20 mol % H included into Si_3N_4.

Interface Impact on Si Nanocrystal Electronic Structure, Connection to Quantum-Chemical Nature of N and O

Below we briefly discuss more detailed data (Fig. S2) corroborating the arguments on interface impact of SiO_2 and Si_3N_4 given in the main article.

The interface charge transfer (ICT) from Si-NCs into interface groups – shown in Fig. S2e by the cumulative Si-NC ionization – would follow a linear behaviour if the interface groups would possess an ionization independent of NC size d_{NC}. Such linear behaviour is shown by the quotient of Si-NC atoms to interface groups (purple data crosses in Fig. S2 e). For strong anions like N or O, the quadratic dependence of transferred charges on NC size massively exceeds the ratio of interface bonds per NC atom [30]. We see from Fig. S2e that the ionization of OH- and NH_2-terminated Si-NCs has a strong quadratic dependence on their diameter. This dependence shows that the ICT is governed by the NC surface, whereby Si-NCs are ionized to their maximum at least up to 1.9 nm NC size. No linear behaviour ($\partial^2(ICT)/\partial(d_{\mathrm{NC}})^2 = 0$) of the ICT can be seen for such NCs which would indicate its saturation, though at a much higher ICT rate as compared to co-valent NC terminations like H. This finding is supported by the nearly constant offsets of E_{HOMO}, E_{HOMO}

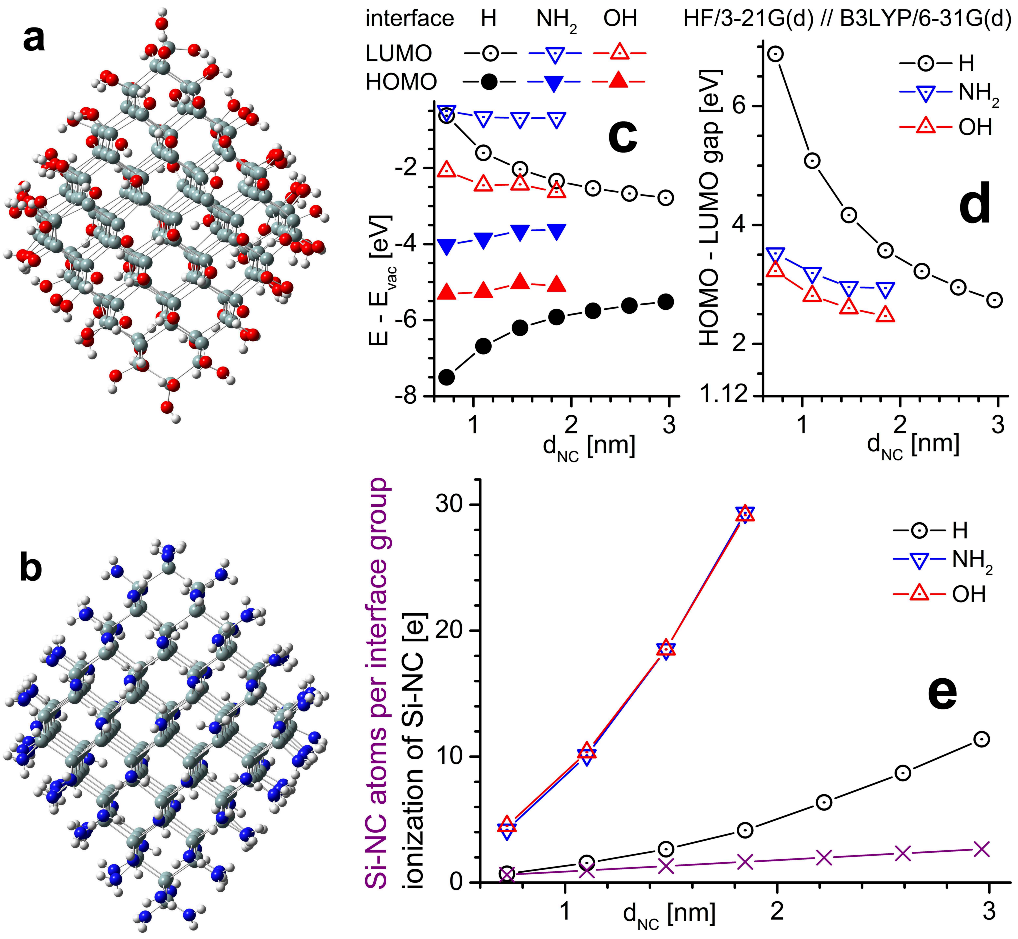

Figure S2: h-DFT Simulation results of Silicon Nanocrystals (Si-NCs). Si-NCs of 1.9 nm size fully terminated with OH groups – $Si_{165}(OH)_{100}$ (a) – and NH_2 groups – $Si_{165}(OH)_{100}$ (b) – after structural optimization, seen along ⟨110⟩ axis to illustrate {111} faceting. Si atom colour is grey, O is red, N is blue and H is white. (c) HOMO and LUMO energy positions referring to vacuum level and (d) resulting fundamental gap of Si-NCs as function of interface termination over NC size. (e) Total Si-NC ionization and ratio of interface groups per Si-NC atom [30] as function of interface termination over NC size.

and E_{gap} between OH- and NH_2-terminated Si-NCs over d_{NC} (Fig. S2c,). Geometrical arguments [22] show that the saturation of the ICT occurs at NC sizes > 7 nm. In this context, we point out that the octahedral {111}-faceted Si-NCs have a *maximum* value of the ratio of NC atoms per interface group as compared to other Si-NCs [30] which leads to the earliest onset of ICT saturation. As a consequence, less symmetric Si-NCs like fin-field-effect transistors (FETs) will have their electronic structure dominated by the ICT to far bigger structure sizes [30].

Normalization of UPS Scans With Further Reference Data

Normalization

Before features such as material-characteristic valence band edges or highest occupied states (HOS) of NWells can be quantitatively evaluated on an energy scale, UPS scans have to get normalized. The normalization of UPS signals has to be carried out at an electron kinetic energy E_{kin} where there are no occupied states and the only contributions to the resulting background signal are events which are comparatively rare to the fundamental photonic excitation of electrons in occupied states. Such processes are free carrier re-absorption and multiple carrier inelastic scattering of primary electrons with high energy secondary electrons and/or other primary electrons [32]. Since a maximum energetic distance of the excited electrons from their origin energy (HOS) enables a maximum de-coupling of the UPS signal from the background, we chose the kinetic energy range $E_{\mathrm{kin}} = [h\nu; h\nu - 0.5\ \mathrm{eV}]$. Normalization is then carried out as follows:

1. All UPS scans are brought onto the same average intensity (counts per second [cps]) in the energy range considered (dash-dotted rectangle in Fig. S3) which requires a weighting factor per scan. This factor is set to one for the reference (Ref-Si-wafer). With the weighting factors, the resulting average cps value is then valid for all samples.

2. The cps offset from zero – here it is 60 cps (dash-dotted line in Fig. S3) – is then substrated from all UPS scans.

The procedure is analog for SiO_2 and Si_3N_4 reference samples but on a different E_{kin} scale due to their different $h\nu$. The scans of these reference samples were normalized to the Si reference in the same manner as explained above using $E_{\mathrm{kin}} = [h\nu; h\nu - 0.5\ \mathrm{eV}] = [20.0; 19.5]$ eV, allowing for a direct comparison on an energy scale relating to E_{vac} (Fig. S4e).

Data of SiO_2 and Si_3N_4 Reference Samples

The valence band energies E_{V} of SiO_2 and Si_3N_4 are located considerably deeper below the vacuum level E_{vac} as compared to the Si-based samples in the main publication, requiring a higher UV photon

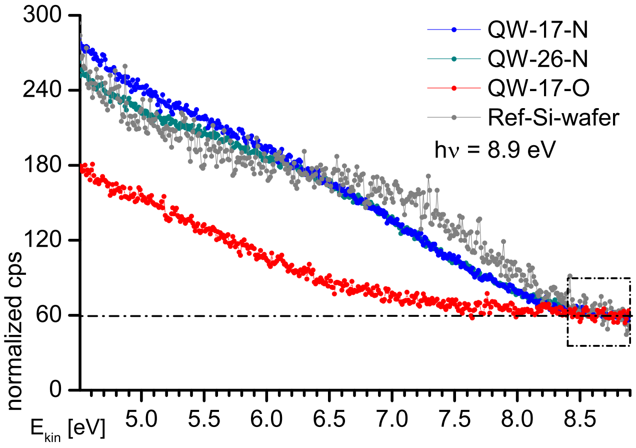

Figure S3: Normalization of UPS signals to enable quantitative comparison of different scans recorded with the same UV photon energy $E(h\nu)$. Data of NWell samples and H-terminated Si wafer as reference for Si NWells are shown here. These scans get assessed at the high end of E_{kin} range - $E_{\text{kin}} = [h\nu; h\nu - 0.5\text{ eV}]$, dash-dot rectangle - where no occupied states exist and only background noise occurs due to comparatively rare events such as free carrier re-absorption and inelastic ≥ 3 particle electron–electron-scattering. This procedure is analog for SiO_2 and Si_3N_4 reference samples. For details of normalization procedure see text.

energy $h\nu$. In contrast to Si-NWells, SiO_2 and Si_3N_4 do not oxidize in air, allowing the respective dielectric to be located at the top of the sample. No delicate balance of dielectric capping layer thicknesses exists as in the case of buried Si-NWell samples. This arrangement allows for maximum UPS signal intensity at elevated UV photon energies. Scans were carried out with $h\nu = 20$ eV. The Si_3N_4, SiO_2 and Si reference samples are shown in Fig. S4a–c.

From Fig. S4d we obtain an offset between the valence band edges of SiO_2 and Si_3N_4 of $\Delta E_V(SiO_2, Si_3N_4) = 2.65$ eV which is in accord with 2.66 ± 0.14 eV found by Keister *et al.* [33]. We obtain a slightly bigger value of $\Delta E_V(SiO_2, Si_3N_4) = 2.75$ eV when we consider the offset between the SiO_2 and the buried Si_3N_4 layer of sample Si-40.25-O.

For obtaining band offsets to bulk Si, we have to relate E_{kin} of the electrons to their binding energy relative to E_{vac} since we cannot directly compare UPS scans carried out with different excitation energies $h\nu$. The binding energies of the valence HOS of bulk materials encountered in the references are identical with E_V so that E_V relative to E_{vac} can be expressed as

$$E_V = E_{\text{kin}}(\text{HOS}) - h\nu \quad . \tag{S1}$$

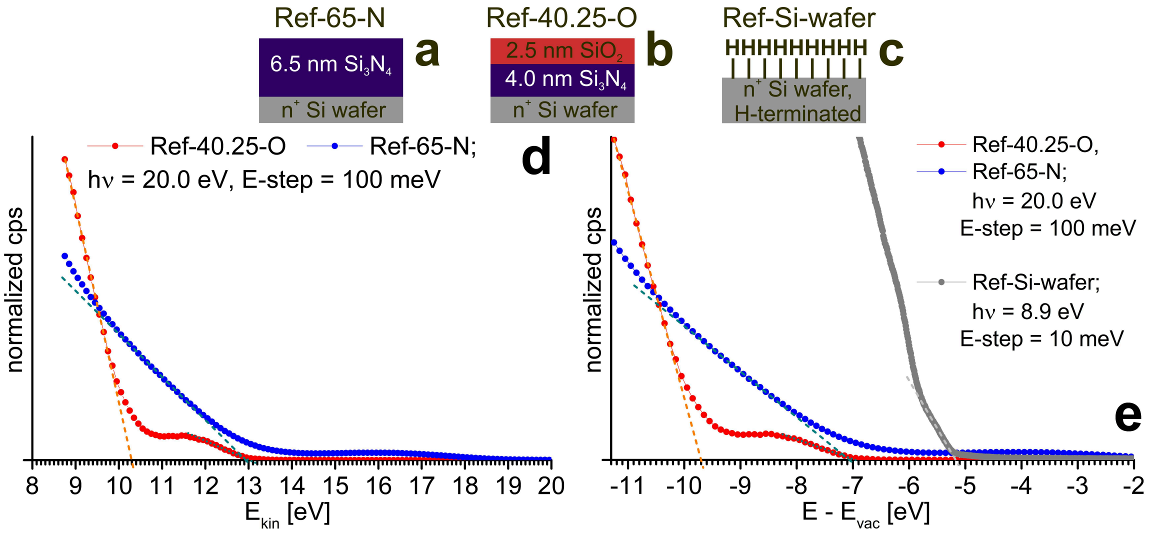

Figure S4: Reference samples and related UPS scans. Reference samples used in our work for Si_3N_4 (a), SiO_2 (b) and Si (c). Respective sample code is shown on top. UPS scans of SiO_2 and Si_3N_4 reference samples as function of kinetic electron energy (d). UPS scans of all three reference samples as a function of binding energy relative to E_{vac} (e). All scans underwent intensity normalization to the Si reference, see text for details.

Fig. S4e shows UPS scans in relation to E_{vac}. The position of the valence band edge of bulk Si is $E_V(\text{Si}) = E_{vac} - 5.17$ eV, matching the electron affinity of bulk Si $X(\text{bulk Si}) = -4.05$ eV minus the Si band gap at $T = 300$ K $E_{gap}(\text{bulk Si}) = 1.12$ eV [34]. We obtain $\Delta E_V(SiO_2, \text{Si}) = 4.53$ eV which closely matches 4.54 ± 0.06 eV found by Keister *et al.* [33]. For the valence band offset of Si_3N_4 to Si we get $\Delta E_V(Si_3N_4, \text{Si}) = 1.88$ eV for 6.5 nm Si_3N_4 (sample Ref-65-N) and $\Delta E_V(Si_3N_4, \text{Si}) = 1.78$ eV for Si_3N_4 buried underneath 2.5 nm SiO_2. Both values once more match the result of Keister *et al.* [33] who found 1.78 ± 0.09 eV.

Signal to Noise Ratio (SNR) of Si-NWells embedded in Si_3N_4

As pointed out in the main article, the SNRs of the 1.7 nm and 2.6 nm Si-NWells embedded in Si_3N_4 are significantly lower as compared to the 1.7 nm Si-NWell embedded in SiO_2. We will investigate the origin of the higher noise level for Si_3N_4-embedded Si-NWells and evaluate the data integrity of these long-term UPS-scans.

There are several factors by which the Si_3N_4 embedding a Si-NWell decreases the SNR as compared to embedding SiO_2 of the same top layer thickness.

The ratio of packing fractions of α-Si_3N_4 to SiO_2 (Cristobalite) is $\mathscr{P}(Si_3N_4/SiO_2) = 1.36$ [31], whereby Si_3N_4 provides 36% more scattering centres per unit volume as compared to SiO_2. This finding is reflected in the normalized cps of the 1.7 nm NWell samples (Fig. 4b in main article): The valence HOS of the Si-NWell in Si_3N_4 has a normalized cps at ca. $1/\mathscr{P}(Si_3N_4/SiO_2) = 1/1.36 = 74\%$ of the cps range where the valence HOS occurs for the Si-NWell embedded in SiO_2. In other words, the 1.5 nm Si_3N_4 top layer is effectively as thick as a 2.0 nm SiO_2 layer as far as inelastic electron scattering is concerned. We can verify this statement for the two 1.7 nm Si-NWells when looking at the normalized signal at the cps ranges of the valence HOS of the respective NWell, yielding 692 ± 70 cps for sample QW-17-N (954 ± 206 cps for sample QW-17-O). From these values we obtain a cps ratio of $692 \pm 70/954 \pm 206 \approx 0.72$ which alignes to $1/\mathscr{P}(Si_3N_4/SiO_2) = 0.74$. The difference in packing fraction is not the only effect to decrease the SNR, though.

It is well known that Si_3N_4 has significantly higher electronic defect densities within its fundamental gap as compared to SiO_2. Indeed, such defects (Urbach tails [34]) can be seen in Fig. 4b of the main article, where the tail states for samples Si-26-N and in particular Si-17-N become much more prominent as compared to sample Si-17-O. The effect of quantum confinement on the SNR is evident from the cps range of the Si-NWell valence HOS of sample QW-26-N which shows a higher SNR at a *lower* intensity of 615 ± 98 cps *although* the NWell is 1.5 times thicker than in sample QW-17-N. Lower quantum confinement of sample QW-26-N shifts its valence HOS further away from the valence band edge of Si_3N_4 and its Urbach tail, resulting in a decreased electronic DOS associated with Si_3N_4 at the energy range where the NWell valence HOS occurs, thereby increasing its SNR.

In addition to higher defect densities within the entire fundamental gap, the energy of the valence band edge of Si_3N_4 with $E_{vac} - 7.05$ eV is much nearer to the valence HOS of Si-NWells as compared to SiO_2 ($E_{vac} - 9.70$ eV, see section). Thereby, the Si_3N_4 valence band DOS and associated Urbach tails provide a yet higher background to the valence HOS signal of the Si-NWells, masking the valence HOS signal of the Si-NWells. The electron affinity X of Si_3N_4 is ca. -1.6 eV, about

twice as low as the value for SiO_2 (-0.8 eV), giving way to a wider energy range of Si_3N_4 where inelastic electron scattering occurs. This contribution further diminishes the cps of excited primary (unperturbed) electrons originating from the valence HOS of the Si-NWell in Si_3N_4.

Another feature contributing to energetic blurring of the valence HOS of Si_3N_4-embedded Si-NWells is their poly-Si nature. Such NWells were processed by depositing and annealing ultrathin amorphous (a-) Si layers, with Si_3N_4 known to restrict a-Si crystallisation as compared to SiO_2 due to its higher Young's modulus and associated strain [35]. Resulting poly-Si NWells have multiple interface orientations and wider deviations in NWell thickness. Although the ICT is far from saturating at ultrathin NWells [22], it induces a minor energetic shift of the valence HOS as a function of interface orientation [30]. Thickness deviations of NWells add to signal blurring as they change the ratio of Si (NWell) atoms per interface bond. This ratio is a key parameter of the ICT impact which induces the energetic shift of the electronic structure of usn-Si [30]. Although the minor thickness deviations encountered here will not induce a major electronic shift, they will suffice to blurr the energetic position of the valence HOS of NWells in the range of a couple of 10 meV. Another obvious reason for signal blurring is the change in quantum confinement with NWell thickness in particuler for the 1.7 nm NWell.

Unfortunately, a further-going quantitative analysis of these phenomena on the SNR of the Si-NWells embedded in Si_3N_4 is not possible nor worthwhile as the exact spot used in UPS would have to be characterized by several other techniques, some of which are destructive to the sample. The solution is to resort to SOI sample processing with Si_3N_4-embedding in analogy to the SOI sample QW-17-O featuring a SiO_2-embedded 1.7 nm Si-NWell consisting of c-Si with {001} interface orientation. Sucessfully processing such Si-NWells in Si_3N_4 is vastly more complex as compared to SiO_2-based SOI processing and a current focus of technological development.

We now turn to the data integrity of the Si_3N_4-embedded samples QW-17-N and QW-26-N. For the c-Si NWell in SiO_2 (sample QW-17-O), we see a clear feature of a valence HOS (see Fig. 4b in main article); this sample is therefore not considered here.

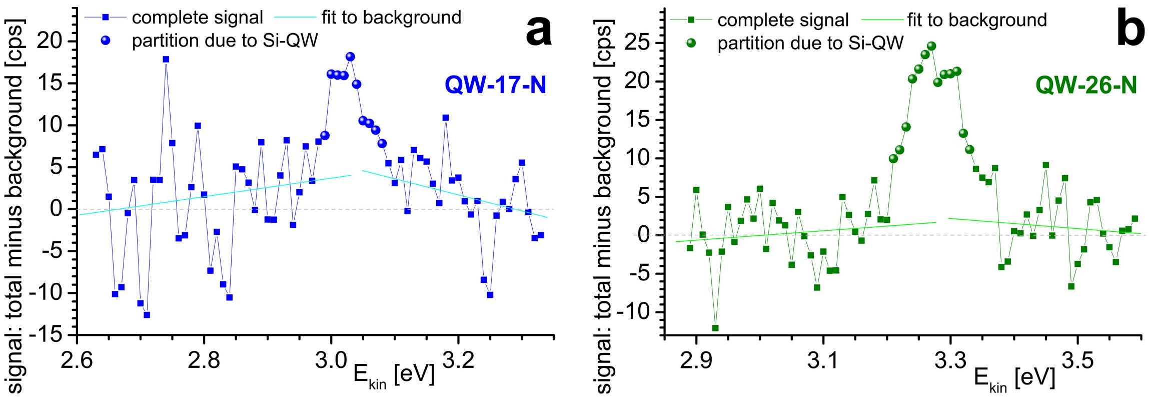

Figure S5: Difference between full UPS signal and fit to amorphous background due to Si_3N_4-embedding of Si-NWells as shown in Fig. 4b of main article for sample QW-17-N having a Si-NWell thickness of 1.7 nm (a) and for sample QW-26-N having a Si-NWell thickness of 2.6 nm (b). Cyan (light green) lines show local linear fits to the signal difference *excluding* the signal originating from the 1.7 nm (2.6 nm) Si-NWell. Data points assigned to the Si-NWells are shown as spheres. For more details see text.

The valence HOS feature of both Si-NWells embedded in Si_3N_4 are fairly weak as compared to the respective feature of sample Si-17-O. We therefore plot the difference between the UPS signal (normalized cps) to the fit of the amorphous background describing the electronic DOS of Si_3N_4 in the vicinity of the valence HOS feature of the respective NWell, see Fig. 4b in main article. In addition, we apply a linear fit to each region of the difference signal above and below the energy range of the valence HOS assigned to the respective Si-NWell. These data are plotted for the 1.7 nm NWell in Fig. S5a and for the 2.6 nm NWell in Fig. S5b.

We first consider sample QW-26-N which shows a clear feature above the two linear fits of the differential signal. This feature can be assigned to the presence of the 2.6 nm NWell straight away as it clearly stands out above the local a-Si_3N_4 background. The feature of the 1.7 nm NWell is just visible above the a-Si_3N_4 background. It contains 10 data points, while fluctuations of the same magnitude due to noise in adjacent energy ranges contains only up to 4 data points above the linear fit. It appears that 1.7 nm is the minimum thickness of a poly-Si NWell in Si_3N_4 to still get detected by long-term UPS of at least 12 h to maximize the SNR of UPS scans. Such valence HOS features could be significantly enhanced if the NWell consisted of c-Si with defined interface orientation embedded in high-quality thermal Si_3N_4, see above.

Derivation of Charge Carrier Densities for Non-Equilibrium Green's Function (NEGF) Transport Simulation of Undoped Si-Nanowire MIS-FET Devices

As mentioned in section , real-space DFT calculations do not provide results with an absolute energy gauge such as the vacuum energy E_{vac} whereby we can only use relative changes in energy such as energy offsets within one or between several approximants calculated with exactly the same DFT computation route. We use results from UPS measurements to obtain absolute energies with respect to E_{vac} and apply fundamental NWire gaps and energy offsets in HOMOs ΔE_{HOMO} obtained from DFT calculations of the NWire approximant (Fig. 5a–c in main publication). The UPS of a 1.7 nm Si-NWell embedded in Si_3N_4 yielded an energy of the HOS of $E_{\mathrm{V}}(\text{Si-NWire}) = E_{\mathrm{vac}} - 5.20$ eV which yields $E_{\mathrm{V}}(\text{Si-NWire}) = E_{\mathrm{V}}(\text{bulk-Si}) - 0.03$ eV. As drain and source electrode materials we use Ni which is known to have an electron work function of $E_{\mathrm{WF}}(\mathrm{Ni}) = 5.15$ eV [36] which is equivalent to $E_{\mathrm{WF}}(\mathrm{Ni}) = E_{\mathrm{V}}(\text{bulk-Si}) + 0.02$ eV. Above values yield to $E_{\mathrm{WF}}(\mathrm{Ni}) - E_{\mathrm{V}}(\text{Si-NWire}) = 0.05$ eV. This energy difference serves to calculate the Fermi-Dirac occupation probability of the Si-NWire valence states with holes:

$$
\begin{aligned}
f_{\mathrm{FD}} &= \frac{1}{1+\exp\left(\frac{E_{\mathrm{WF}}(\mathrm{Ni}) - E_{\mathrm{V}}(\text{Si-NWire})}{k_{\mathrm{B}}T}\right)} \\
&= 0.1263 \quad ,
\end{aligned}
\tag{S2}
$$

with $k_{\mathrm{B}}T$ presenting the thermal energy at $T = 300$ K. This f_{FD} value applies to all 121 Si atoms within the NH_2-covered NWire section of the $Si_{233}(NH_2)_{87}(OH)_{81}$ approximant; $\mathcal{N}_{\mathrm{Si}}(\mathrm{NH_2\text{-}term}) = 121$. In other words, we get one hole for every $\mathcal{N}_{\mathrm{Si}}(\mathrm{NH_2\text{-}term})/f_{\mathrm{FD}} = 958$ atoms. Relating this ionization probability to the atomic volume density of Si, $N_{\mathrm{Si}} = 5.16 \times 10^{22}$ atoms/cm^{-3}, we arrive at a hole density within the Si_3N_4-covered NWire section of $p = N_{\mathrm{Si}} \times f_{\mathrm{FD}}/\mathcal{N}_{\mathrm{Si}}(\mathrm{NH_2\text{-}term}) = 5.36 \times 10^{19}$ cm^{-3}.

The hole density on the OH-terminated section of the $Si_{233}(NH_2)_{87}(OH)_{81}$ approximant follows

the reasoning outlined above with two different parameters: $\mathscr{N}_{\mathrm{Si}}$(OH-term) = 112 and E_{WF}(Ni) − E_{V}(Si-NWire) = 1.12 eV, yielding to $f_{\mathrm{FD}} = 1.532 \times 10^{-19}$ and $p = 71$ cm^{-3}. In contrast to what this very low value suggests, it is quite stable since it reflects the energy offset between Si_3N_4- and SiO_2-covered Si-NWire sections. This value of p is fairly robust against carrier diffusion processes due to the dominance of O onto the channel volume of the Si-NWire (Fig. 5a in main publication). It can be controlled by applying a gate-source voltage counter-acting the ICT from the NWire to the O atoms within SiO_2.

Experimental input on Si-NWires with diameters of 1.7 nm as used in NEGF-Simulations from UPS-scans would provide the ideal energy calibration for the energetic consideration above. However, the sample volume would be too small to get useable results. With the ratio of quantum numbers to thickness of NWires vs. NWells, we could get a good estimate of the NWire HOSs relative to E_{vac}. The prospective Si-NWell would need a thickness of $1/\sqrt{2}$ of the Si-NWire diameter due to the ground state of the system having one quantum number for a QWell $\langle 1 \rangle$ and two quantum numbers $\langle 1|1 \rangle$ for a QWire [37]. This would mean we had to measure a Si-NWell of 1.7 nm $\times 1/\sqrt{2} = 1.2$ nm thickness which is not feasible due to the NWell HOS signal being buried by noise, see section . In other words, the UPS data obtained from the 1.7 nm Si-NWell embedded in Si_3N_4 currently provides us with the best practicable experimental input to a 1.7 nm Si-NWire, with the prospect to characterize yet thinner Si-NWells if SOI-processed Si_3N_4-based samples are at hand.

For the simulations of FET devices based on ultrathin Si-NWires with appropriate SiO_2 and Si_3N_4 coatings to realize ICT we solved self-consistently Poisson's and Schrödinger's equation using the NEGF formalism. For minimizing computational cost, a one-dimensional finite difference, effective mass model for the electrostatics and for the NEGF is employed which has proven to provide adequate results for devices based on NWires with ultrathin diameters [39,41]. As has been shown in [42], the electrostatics of a nanowire transistors can be described well by a modified 1D equation of the following form:

$$\frac{d^2\Phi_{\mathrm{f}}}{dx^2} - \frac{\Phi_{\mathrm{f}} - \Phi_{\mathrm{g}} + \Phi_{\mathrm{bi}}}{\lambda^2} = \frac{e(\rho(x) \pm N)}{\varepsilon_0 \varepsilon_{\mathrm{Si}}} \tag{S3}$$

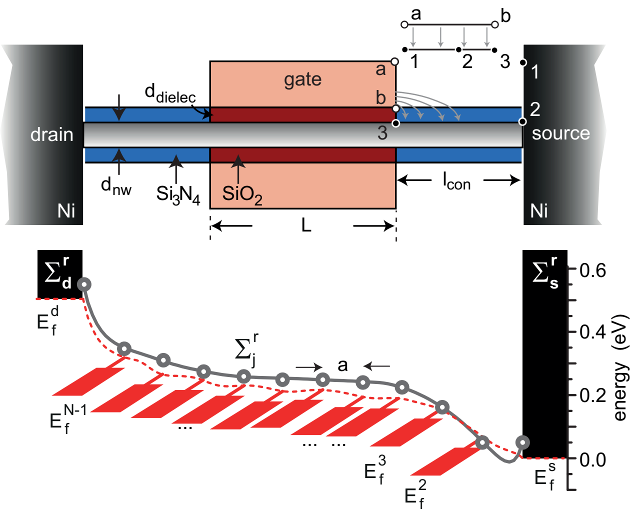

Figure S6: Schematics of the nanowire device under consideration. A nanowire of diameter d_{nw} is surrounded by Si_3N_4 within the gate area and covered by SiO_2 in the gate underlap region (length l_{con}). The nanowire is contacted with nickel contacts; fringing fields are taken into consideration with conformal mapping [38,39]. The lower panel shows the valence band through the device (solid dark gray line) and the spatial dependence of the quasi-Fermi level (red dashed line). Buettiker probes (red contacts) are attached at each finite difference grid size [40]; the Buettiker probes are isolated virtual contacts whose (quasi-)Fermi level is found self-consistently based on the requirement that each Buettiker probe carries zero net current.

where Φ_f, Φ_g and Φ_{bi} are the surface potential, the gate potential and the built-in potential, respectively, and $\rho(x)$ is the density of free carriers. The length λ represents a screening length for potential variations and its functional form depends on the system under consideration. In the present case of a gate-all-around nanowire FET we approximate the actual cylindrical device layout with a geometry consisting of four gates placed around the nanowire and hence $\lambda = \sqrt{\frac{\varepsilon_{Si}}{\varepsilon_{Si_3N_4}} d_{ch} d_{dielec}/4}$ [39]. Within the un-gated underlap region of length l_{con}, fringing fields of the gate need to be taken into consideration. To this end, the area bounded by the points 1,2,3 and a, b in cartesian coordinates is conformally mapped onto a parallel plate capacitor (see Fig. S6) [38]. On transforming back the electric field lines, a spatially dependent $d_{dielec}(x)$ and hence $\lambda(x) = \sqrt{\frac{\varepsilon_{Si}}{4\varepsilon_{air}} d_{dielec}(x) d_{nw}}$ are obtained that allow using Eqn. (S3) for the entire device; note that for simplicity we neglected the small contribution of the SiO_2-layer to the effective dielectric constant within the gate underlap region.

The charge in and current through the device is computed self-consistently assuming one-dimensional electronic transport through the nanowire as appropriate for the dimensions of the

nanowire considered here. An effective mass approximation is used for the conduction and valence band and the complex band structure within the band gap is taken into consideration via Flietner's dispersion relation [43]. The magnitude of the band gap is take from the h-DFT calculations (see above). In order to describe inelastic carrier scattering, Buettiker probes are attached to each finite difference site (illustrated with the circles in Fig. S6) as displayed in the lower panel of Fig. S6. A mean free path of $l_{\mathrm{mfp}} = 5\,\mathrm{nm}$ has been assumed giving rise to the quasi-Fermi level distribution shown in Fig. S6 (red dashed line).

References

1. J. S. Binkley, J. A. Pople, W. J. Hehre, *J. Am. Chem. Soc.* **1980**, *102*, 939.

2. K. D. Dobbs, W. J. Hehre, *J. Comp. Chem.* **1987**, *8*, 880.

3. J. B. Foresman, A. Frisch, *Exploring Chemistry with Electronic Structure Methods (2. Ed.)*, Gaussian Inc., Wallingford, CT, USA (1998).

4. D. König, J. Rudd, G. Conibeer, M. A. Green, *Phys. Rev. B* **2008**, *78*, 035339.

5. EPAPS document to [4].

6. C. B. R. dos Santos, C. C. Lobato, J. B. Vieira, D. S. B. Brasil, A. U. Brito, W. J. C. Macêdo, J. C. T. Carvalho, J. C. Pinheiro, *Comp. Molec. Biosci.* **2013**, *3*, 66.

7. S. Kümmel, L. Kronik, *Rev. Mod. Phys.* **2008**, *80*, 3.

8. 2008 ARC Photovoltaics Centre of Excellence Annual Report, **2008**, p. 86 hardcopy [p. 88 pdf file].

9. D. König, S. Gutsch, H. Gnaser, M. Wahl, M. Kopnarski, J. Göttlicher, R. Steininger, M. Zacharias, D. Hiller, *Sci. Rep.* **2015**, *5*, 09702.

10. D. König, *The Introduction of Majority Carriers into Group IV Nanocrystals (Chapter 7)*, pp. 203-254, in Valenta, J., Mirabella, S. (Eds.) *Nanotechnology and Photovoltaic Devices*, Pan Stanford Publishing, Singapore (2015).

11. D. König, J. Rudd, G. Conibeer, M. A. Green, *Mat. Sci. Eng. B* **2009**, *159-160*, 117.

12. R. L. Mozzi, B. E. Warren, *J. Appl. Cryst.* **1969**, *2*, 164.

13. J. R. G. Da Silva, D. G. Pinatti, C. E. Anderson, M. L. Rudee, *Phil. Mag.* **1975**, *31*, 713.

14. L. W. Hobbs, C. E Jesurum, B. Berger, *The Topology of Silica Networks (Chapter 1)*, pp. 3-47, in R. A. B. Devine, J.-P. Durand, E. Dooryhée (Eds.) *Structure and Imperfections in Amorphous and Crystalline Silicon Dioxide*, Wiley & Sons, Chichester (2000).

15. R. Hezel, C. Jaeger, *J. Electrochem. Soc.* **1989**, *136*, 518.

16. L. Cai, A. Rohatgi, S. Han, G. May, M. Zou, *J. Appl. Phys.* **1989**, *83*, 5885.

17. A. Iqbal, W. B. Kackson, C. C. Tsai, J. W. Allen, C. W. Bates Jr., *J. Appl. Phys.* **1987**, *61*, 2947.

18. T. Aiyama, T. Fukuyama, K. Niihara, T. Hirai, K. Suzuki, *J. Non-Cryst. Sol.* **1979**, *33*, 131.

19. M. Misawa, T. Fukunaga, K. Niihara, T. Hirai, K. Suzuki, *J. Non-Cryst. Sol.* **1979**, *34*, 313.

20. S. Hasegawa, L. He, Y. Amano, T. Inokuma, *Phys. Rev. B* **1993**, *48*, 5315.

21. F. Rissner, D. A. Egger, A. Natan, T. Körzdörfer, S. Kümmel, L. Kronik, E. Zojer, *J. Am. Chem. Soc.* **2011**, *133*, 18634.

22. D. König, D. Hiller, S. Gutsch, M. Zacharias, *Adv. Mater. Interfaces* **2014**, *1*, 1400359.

23. R. W. Hunter, S. A. Malo, *J. Phys. Chem. Sol.* **1969**, *30*, 2739.

24. D. A. Stephenson, N. J. Binkowski, *J. Non-Cryst. Sol.* **1976**, *22*, 399.

25. R. J. Powell, G. F. Derbenwick, *IEEE Tr. Nucl. Sci.* **1971**, *18*, 99.

26. D. H. DiStefano, D. E. Eastman, *Sol. Stat. Comm.* **1971**, *9*, 2259.

27. H. Ibach, J. E. Rowe, *Phys. Rev. B* **1974**, *10*, 710.

28. R. J. Powell, M. Morad, *J. Appl. Phys.* **1978**, *49*, 2499.

29. H. Kurata, H. Masataka, Y. Osaka, *Jap. J. Appl. Phys.* **1981**, *20*, L811.

30. D. König, *AIP Adv.* **2016**, *6*, 085306.

31. A. F. Holleman, E. Wiberg, N. Wiberg, *Lehrbuch der Anorganischen Chemie (101. Ed.)*; Walter deGruyter: Berlin, 1995; (in German).

32. S. Suga, A. Sekiyama, *Photoelectron Spectroscopy – Bulk and Surface Electronic Structures*, Springer Series in Optical Sciences, Vol. 176, Springer Press, Heidelberg, 2014.

33. J. W. Keister, J. E. Rowe, J. J. Kolodziej, H. Niimi, T. E. Madey, G. Lucovsky, *J. Vac. Sci. Tech. B* **1999**, *17*, 1831.

34. K. W. Böer, *Survey of Semiconductor Physics, Vol. 1*. (Van Nostrand Reinhold, New York, 1990).

35. S. R. Elliot, *The Physics and Chemistry of Solids*. (Wiley, Chichester, 1998).

36. S. Hala, T. Durakiewicz, *J. Phys.: Cond. Matter* **1998**, *10*, 10815.

37. J. Singh, *Quantum Mechanics – Fundamentals and Applications to Technology*, Wiley & Sons (New York, 1997).

38. S. Xiong, T.-J. King, J. Bokor, *Semicond. Sci. Technol.* **2005**, *20*, 652.

39. J. Knoch, J. Appenzeller, *IEEE Electron. Dev. Lett.* **2010**, *31*, 305.

40. R. Venugopal, M. Paullson, S. Goasguen, S. Datta, M. S. Lundstrom, *J. Appl. Phys.* **2003**, *93*, 5613.

41. J. Appenzeller, J. Knoch, M. T. Björk, H. Schmid, H. Riel, W. Riess, *IEEE Tr. Electron. Dev.* **2008**, *55*, 2827.

42. C. P. Auth, J. D. Plummer, *IEEE Electron. Dev. Lett.* **1997**, *18*, 74.

43. H. Flietner, *Phys. Stat. Sol.* **1972**, *54*, 201.
